# Characterization of MADS-Box Gene Family in *Isatis indigotica* and Functional Study of *IiAP1* in Regulating Floral Transition and Formation

**DOI:** 10.3390/plants14010129

**Published:** 2025-01-04

**Authors:** Yanqin Ma, Yanhong Lan, Ju Li, Haicheng Long, Yujie Zhou, Zhi Li, Mingjun Miao, Jian Zhong, Haie Wang, Wei Chang, Ziqin Xu, Liang Yang

**Affiliations:** 1 Horticulture Research Institute, Sichuan Academy of Agricultural Sciences, Chengdu 610066, China; dora0514@sina.cn (Y.M.); yanhong_lan2022@163.com (Y.L.); dandelionlj@126.com (J.L.); longhaicheng2024@126.com (H.L.); 15680897967@sina.cn (Y.Z.); lz20031977@126.com (Z.L.); 7200175@uestc.edu.cn (M.M.); zhongjian15@scsaas.cn (J.Z.); hewang-scnky@scsaas.cn (H.W.); 2 Key Laboratory of Horticultural Crops Biology and Germplasm Enhancement in Southwest Regions, Ministry of Agriculture and Rural Affairs of the P.R. China, Chengdu 610066, China; 3 Vegetable Germplasm Innovation and Variety Improvement Key Laboratory of Sichuan Province, Chengdu 610066, China; 4 Key Laboratory of Resource Biology and Biotechnology in Western China (Ministry of Education) Provincial Key Laboratory of Biotechnology, College of Life Sciences Northwest University, Xi’an 710069, China; ziqinxu@nwu.edu.cn; 5 Sichuan Institute of Edible Fungi, Chengdu 610066, China; changwei972@126.com

**Keywords:** *Isatis indigotica*, MADS-box gene, transcription factors, *IiAP1*, floral transition, floral formation

## Abstract

In flowering plants, MADS-box genes play regulatory roles in flower induction, floral initiation, and floral morphogenesis. *Isatis indigotica* (*I*. *indigotica*) is a traditional Chinese medicinal plant. However, available information concerning MADS-box genes in *I*. *indigotica* is insufficient. Based on the sequencing data of the *I*. *indigotica* transcriptome, we identified MADS-box gene-encoding transcription factors that have been shown to play critical roles in developmental processes. In this study, 102 *I*. *indigotica* MADS-box genes were identified and categorized into type I (Mα, Mβ, and Mγ) and type II (MIKC^C^ and MIKC*) subfamilies. IiMADS proteins in the same cluster had similar motifs and gene structures. In total, 102 IiMADS-box genes were unevenly distributed across seven chromosomes. *APETALA1* (*AP1*) encodes a MADS-box transcription factor which plays a pivotal role in determining floral meristem identity and also modulates developmental processes within the perianth. We then selected *IiAP1* for functional studies and found that it is localized to the nucleus and highly expressed in inflorescence, sepals, and petals. The ectopic expression of *IiAP1* in Arabidopsis resulted in early flowering and abnormal development of floral organs. Taken together, this research study carried out a systematic identification of MADS-box genes in *I*. *indigotica* and demonstrated that *IiAP1* takes part in the regulation of floral transition and formation.

## 1. Introduction

The flowers of angiosperms typically possess four floral organs, including sepals, petals, stamens, and carpels [1,2]. Previous studies have demonstrated that floral homeotic genes play a role in determining the identity of floral organs [3]. The genes were classified into three categories according to the ABC model [3]. In Arabidopsis, the A-function genes *APETALA1* (*AP1*) and *AP2* regulate the specification of sepals; the A-function gene *AP1*, in conjunction with the B-function genes *AP3* and *PISTILLATA* (*PI*), is involved in the determination of petals; the B-function genes *AP3* and *PI*, together with the C-function gene *AGAMOUS* (*AG*), characterize the formation of stamens; the C-function gene *AG* facilitates the development of pistils [3]. In addition to uncovering the functional roles of *SEED STICK* (*STK*), *SHATTERPROOF1* (*SHP1*), and *SHP2* in the development of ovules, as well as the *SEPALLATA* (*SEP*) genes in the differentiation process of all floral organs, subsequent advancements were carried out to expand the ABC model into the ABCD and ABCDE models [4,5]. Based on the ABCDE model, the floral quartet model has been proposed, which suggests that floral organ formation is regulated by tetramers composed of MADS transcription factors [6]. All A, B, C, D, and E class genes, with the exception of the putative A-class gene *APETALA2* (*AP2*), encode MADS-box proteins [7,8].

In most eukaryotic organisms, the MADS-box proteins play regulatory roles in growth, development, and signal transduction processes [9], and the MADS-box gene can be categorized as either type I (Mα, Mβ, and Mγ) or type II (MIKC^c^ and MIKC*) [10,11,12]. The MIKC^c^-type MADS-box transcription factors have a highly conserved structure, comprising the MADS domain at the N-terminus (M), the intervening domain (I), the keratin-like domain (K), and the C-terminal domain (C) [13]. The M domain is the most conserved region within MIKC-type MADS transcription factors. It is responsible for binding to the CArG-box elements in target genes, while also facilitating dimerization and interaction with cofactors [13]. The I domain exhibits lower conservation compared to the MADS-box and K domains, which mediate the dimerization of these proteins [14]. The K domain exhibits a coiled-coil structure and participates in protein–protein interactions [15,16], whereas the C domain, known for its lower conservation level, plays a crucial role in protein–protein interactions and the regulation of transcriptional activation [17].

The characterization of various MADS-box genes has been a focal point in the plant biology community due to their crucial roles in plant development, especially in floral organogenesis. Systematic genome-wide analysis of the MADS-box gene family has been extensively performed in numerous plant species including *Arabidopsis thaliana* [12], rice (*Oryza sativa*) [18], maize (*Zea mays*) [19], Sacred lotus (*Nelumbo nucifera*) [20], Wheat (*Triticum aestivum*) [21,22], chrysanthemum (*Chrysanthemum nankingense*) [23], *Camellia chekiangoleosa* [24], *Lonicera japonica* [25], barley (*Hordeum vulgare* L.) [26], and Blueberry (*Vaccinium* spp.) [27].

*Isatis indigotica* Fortune (*I. indigotica* Fort.), a biennial herbaceous plant in the cruciferous family, is commonly referred to as indigo root. This traditional Chinese medicinal plant is valued for its therapeutic properties, with both its roots and leaves utilized for their heat-clearing, detoxifying, blood-cooling, and sore throat-relieving effects [28]. Several essential MADS-box homologs have been identified in *I*. *indigotica*, such as *AP3* [29], *AGL6* [30], *FRUITFUL*(*FUL*) [31], *PI* [29], *SEP1* [32], *SEP2* [33], *SEP3* [34], *SEP4* [35], *SHP2* [36], and *SHORT VEGETATIVE PHASE* (*SVP*) [37]. However, there is currently a lack of comprehensive reports about the identification and function of MADS-box family genes in *I. indigotica*. In this study, RNA-seq was conducted on *I. indigotica* floral tissue. In total, 102 potential MADS-box genes have been identified, and an analysis of candidate gene phylogeny and structure is carried out. We chose the *IiAP1* gene as our target gene for further investigation and found that *IiAP1* is expressed in the inflorescence, sepals, and petals and the encoding protein is localized in the nucleus. The ectopic expression of *IiAP1* in Arabidopsis not only promotes flowering but also affects the development of floral organs. Our research facilitated the investigation of *I. indigotica* gene families and yielded insights into the functions of *I. indigotica* genes. The manipulation of MADS-box gene expression through genetic engineering techniques can effectively extend the vegetative growth period, thereby resulting in increased yields of roots and leaves and enhancing the medicinal value of *I. indigotica*.

## 2. Results

### 2.1. Identification of the IiMADS Genes in I. indigotica

We identified 102 MADS-box genes, namely *IiMADS1*-*IiMADS102*, from *I. indigotica* (File S1). The physical and chemical properties of these MADS-box genes were analyzed (Table S1). The amino acid lengths ranged from 80 (IiMADS73) to 369 (IiMADS90) aa. The molecular weights (MWs) ranged from 9.473 (IiMADS32) to 42.350 (IiMADS6) kDa, and the theoretical isoelectric point (pIs) changed from 4.36 (IiMADS3) to 10.98 (IiMADS73). Subcellular localization analysis showed that all IiMADS proteins were located in the nucleus (Table S1). In addition, the IiMADS gene family’s signal peptide was predicted using SignalP (version v5.0b) software. The results showed that the presence of signal peptides was not predicted in the entire IiMADS gene family. The instability index ranged from 31.38 (IiMADS22) to 69.15 (IiMADS54), the aliphatic index ranged from 55.78 (IiMADS11) to 100 (IiMADS100), and the grand average of hydropathicity ranged from −1.04 (IiMADS54) to −0.231 (IiMADS7), indicating that all IiMADSs were hydrophilic proteins.

### 2.2. Phylogenetic Investigation of IiMADS Genes

**Figure 1:**
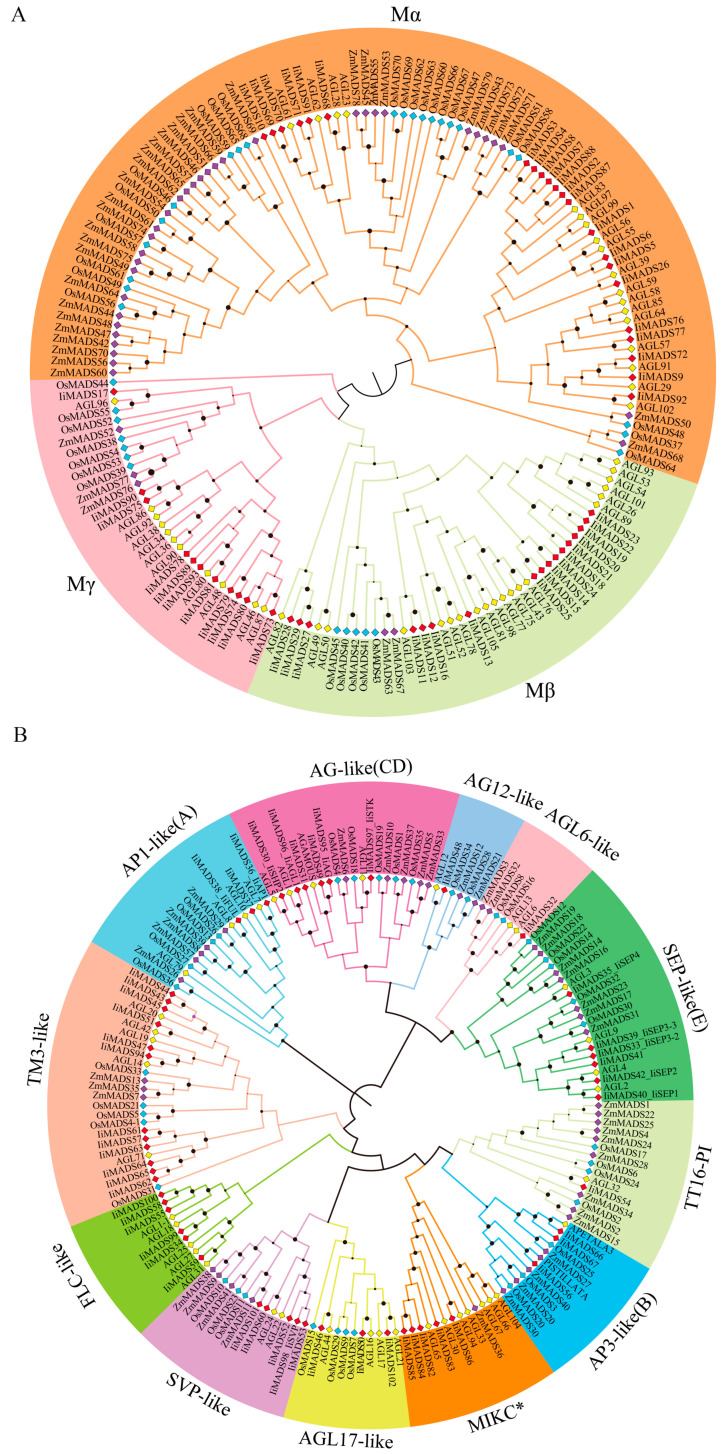
Phylogenetic tree of MADS-box protein sequences in *I. indigotica*, *Arabidopsis thaliana*, *Oryza sativa*, and *Zea mays*. (**A**): Phylogenetic tree of type I MADS-box protein in *I. indigotica*, *Arabidopsis thaliana*, *Oryza sativa*, and *Zea mays*, (**B**): Phylogenetic tree of type II MADS-box protein in *I. indigotica*, *A. thaliana*, *Oryza sativa*, and *Zea mays*. The phylogenetic tree was built with MEGAX and then edited and beautified with Evolview, an online tool.

**Figure 2:**
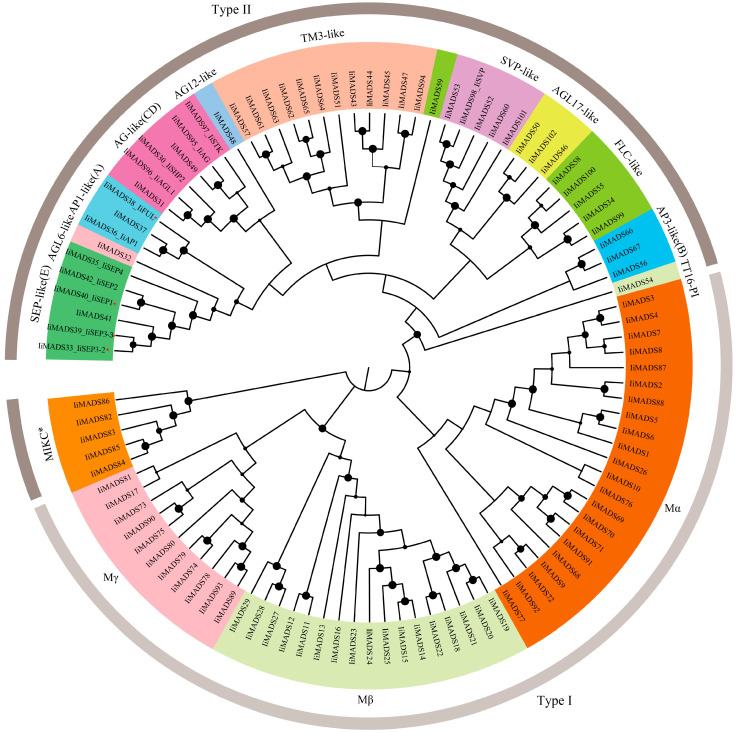
The protein sequence analysis of IiMADS proteins. The Asterisks denote publicly accessible IiMADS proteins. IiMADS38-IiFUL (GenBank accession number: BBB04554.1), IiMADS40-IiSEP1 (GenBank accession No. BBJ25277.1), IiMADS33-IiSEP3-2 (GenBank accession: XDF38148.1), and IiMADS39-IiSEP3-3 (GenBank accession: XDF38149.1).

In order to more systematically understand the phylogenetic relationship of the IiMADS gene family, we utilized MEGAX v11.0 software to construct a phylogenetic tree based on the amino acid sequences of MADS proteins derived from *I. indigotica* as well as various plant species (Arabidopsis, maize, tomato, wheat, and rice). All IiMADS proteins were divided into 15 clades marked with different colors and classified into MADS protein type I and MADS protein type II (Figure 1). The number of IiMADS proteins in type II (52) is more than that in type I (50) (Figure 2). Type I proteins were divided into three subfamilies: Mα, Mβ, and Mγ (Figure 1A and Figure 2). The maximum number of IiMADS found in the Mα subfamily contained 22 IiMADS proteins, while Mβ and Mγ branches contained 17 and 11 IiMADS proteins, respectively (Figure 1A and Figure 2). A total of 12 subfamilies were found in the IiMADS genes type II protein, including the following: AP1-like (A), AP3-like (B), AG-like (C/D), SEP-like (E), SVP-like, FLC-like, AGL6-like, AGL12-like, AGL17-like, TM3-like, TT16/PI, and MIKC* (Figure 1B and Figure 2). In particular, the SEP-like (E) subclass exhibited the highest abundance of IiMADS genes and was significantly expanded in *I. indigotica* in contrast to Arabidopsis. IiAP1 and liFUL were found in the AP1-like (A) subfamily. These results suggested that they may have similar functions, such as playing a central role in the regulatory network of plant floral induction and determining the formation of floral organs. Similar findings were also found in other plants [38,39] (Figure 1B).

### 2.3. Analysis of Gene Structure, Conserved Motifs, and Cis-Acting Elements in the IiMADS Genes

**Figure 3:**
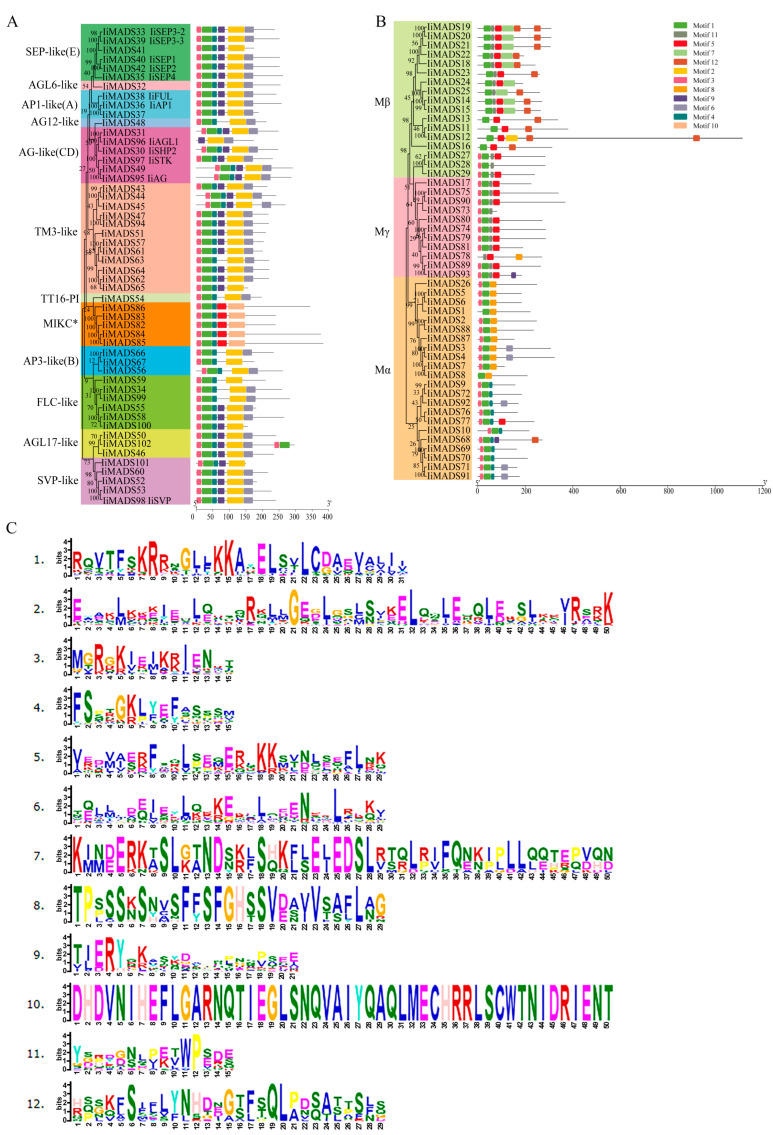
Motif of the *I. indigotica* MADS gene family. (**A**): Motif of the type II IiMADS gene family, (**B**): Motif of the type I IiMADS gene family, (**C**): the different motif. Colored boxes indicated different conserved motifs, while the black lines represent sequences in which no MEME motifs were detected.

**Figure 4:**
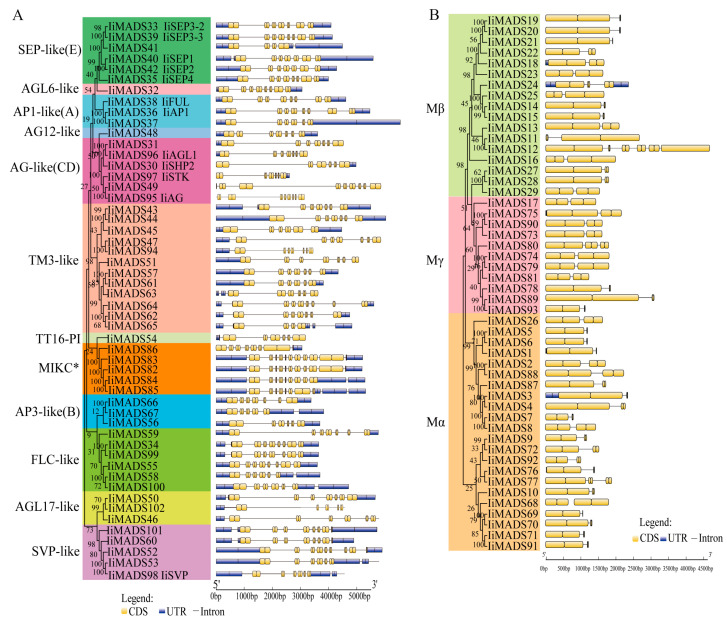
Gene structure of the *I. indigotica* MADS gene family. (**A**): Gene structure of the type II IiMADS gene family, (**B**): Gene structure of the type I IiMADS gene family. Exons were shown as yellow boxes, introns are shown as black lines, and UTRs were shown as blue boxes.

To delve deeper into the structural diversity, similarity, and evolutionary relationships of IiMADS genes, an analysis of conserved motifs and intron-exon arrangements was conducted (Figure 3 and Figure 4). The results showed that the IiMADS gene family contained a total of 12 motifs, with each of the 102 IiMADS genes possessing 2 to 8 motifs. Notably, all genes contained motif 1, indicating its crucial role in the IiMADS gene family. Motif 10 was exclusively present in the MIKC* clade, motif 12 was specific to the Mβ clade, and motif 8 was found only in Mα. Additionally, motif 9 is present solely in the MIKC^C^ subfamily (Figure 3). Furthermore, all family genes contained introns, ranging in number from 1 to 8, indicating a significant variation in intron density within *I. indigotica* (Figure 4). Overall, when considering the evolutionary tree of the IiMADS gene family, genes with similar motif structures and gene structures tended to be grouped within the same subclass of the phylogenetic tree.

**Figure 5:**
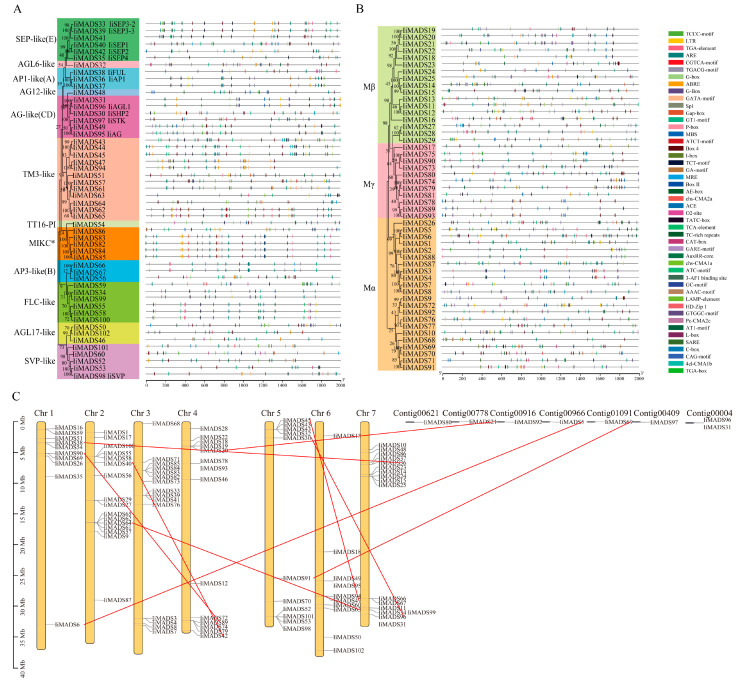
The cis-acting element combination and chromosomal distribution of the *I. indigotica* MADS gene family. (**A**,**B**): The cis-acting element combination of the *I. indigotica* MADS gene family, (**C**): Chromosomal distribution and collinearity analysis of the IiMADS gene family. The Chr number is indicated at the top of each chromosome; the scale is in megabases (Mb); the red lines represent the connections between homologous gene pairs.

The results of the *cis*-acting element analysis of IiMADS family genes (Figure 5A,B) showed that the promoters of the IiMADS family genes contained meristem expression (CAT-box), zein metabolism regulation (O_2_-sites), photoresponsive elements (I-box, CAG-motif, G-box, etc.), hormone-responsive elements for auxin(TGA-box and AuRR-core), jasmonic acid (CGTCA-motif and TGACG-motif), abscisic acid (ABRE), and gibberellin (TATC-box, P-box and GARE-motif), as well as elements related to low temperatures and defense responses. The IiMADS genes exhibited a rich variety of *cis*-acting elements, with photoresponsive elements being relatively abundant, potentially playing an important role in plant growth and development. Additionally, the IiMADS genes were enriched with *cis*-acting elements related to stress responses, including low-temperature response (LTR) elements and drought response elements (MBS), indicating that the IiMADS genes may confer resistance to multiple stresses and suggesting functional diversity. In summary, IiMADS genes may play a crucial role in stress response and the growth and development of *I. indigotica* plants, particularly during endosperm development.

### 2.4. Chromosomal Distribution and Collinearity Analysis of IiMADS Genes

Based on *I. indigotica* genome annotation [40], a chromosomal localization and gene collinearity map of IiMADS genes was constructed (Figure 5C). The results showed that 102 IiMADS genes were unevenly distributed across seven chromosomes, with 8 genes located on seven Contigs. An intraspecific collinearity analysis of the *I. indigotica* MADS gene family was performed using MCScanX (http://chibba.pgml.uga.edu/mcscan2/MCScanX.zip, accessed on 4 January 2012) (Figure 5C), revealing nine pairs of homologous genes. The red lines represent nine groups of IiMADS duplicated gene pairs, and the Ka and Ks values, as well as their ratios (Ka/Ks), for each duplicated gene pair, are listed in Table 1. The Ka values of the duplicated gene pairs ranged from 0.0194865 (IiMADS90/IiMADS42) to 0.0921623 (IiMADS91/IiMADS69), and the Ks values ranged from 0.195654 (IiMADS5/IiMADS6) to 0.511771 (IiMADS40/ IiMADS89). The Ka/Ks ratio, an indicator of evolutionary selection pressure, was less than 1.00 for all pairs, indicating that purifying selection occurred during the evolution of *I. indigotica*.

### 2.5. Protein Network Interaction of IiMADS Genes

In order to thoroughly analyze the characteristics of the IiMADS proteins in *I. indigotica*, the protein functional relationships, and proteins in the protein–protein interaction (PPI) network of the IiMADS gene family were predicted using the String database with Arabidopsis proteins as a reference. The results showed (Figure S1) that 58 interaction relationships were identified among 51 IiMADS proteins, and most interactions occurred between gene families. Most proteins in the PPI network were related to flowering regulation (IiMADS35, IiMADS38, and IiMADS45, etc.), floral organ development (IiMADS40, IiMADS42, and IiMADS56, etc.), participation in post-embryonic plant organ development (IiMADS41 and IiMADS56), and maintenance of the floral meristem identity (IiMADS35 and IiMADS94). Some proteins were also involved in plant ovule development (IiMADS30, IiMADS40, IiMADS54, etc.).

### 2.6. Isolation of IiAP1 from I. indigotica

**Figure 6:**
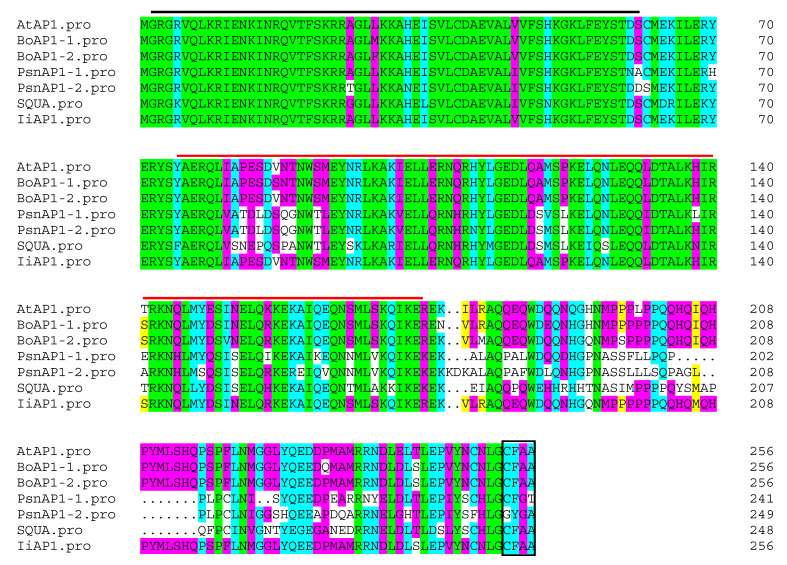
Multiple alignments of the amino acid sequences of AP1 homologous proteins. The same amino acids are shown in green. The identities of amino acids, which are more than 75%, 50%, and 30%, are shown in blue, purple, and yellow, respectively. Black frames represent the CFAA motif. The black line represents the MADS-box domain; the red line represents the K domain. *Arabidopsis thaliana* AP1 (NP-177074); BoAP1-1, MADS-box protein AP1-a of *Brassica oleracea* var. *botrytis* (CAD47853); BoAP1-2, homeotic protein boi1AP1 of *Brassica oleracea* var. *italica* (AAB08875); PsnAP1-1, *Populus simonii* × *Populus nigra* APETALA1-like MADS-box AP1-1 (KC866354.1); PsnAP1-2, *Populus simonii* × *Populus nigra* APETALA1-like MADS-box AP1-2 (KC866355.1); SQUA, ortholog of AP1 in *Antirrhinum majus* (CAA45228.1); IiAP1, woad AP1 determined in the present study.

In Arabidopsis, the *APETALA1* (*AP1*) gene is essential for establishing a floral meristem identity, promoting the development of sepals and petals, and preventing flower formation within the sepal axils. In this study, the 3′- and 5′-unknown cDNA regions of *IiAP1* were determined via RACE methods. The 1002 bp *IiAP1* cDNA contained an open reading frame of 771 bp and encoded a 256 amino acid protein (Figure S2). IiAP1 is a typical MIKC-type MADS protein that contains four structural domains, namely the highly conserved MADS-box domain (1~60 aa), I domain (61~74 aa), K domain (75~174 aa), and C-terminal domain (175–256 aa). Sequence alignment revealed that the IiAP1 protein shared a high degree of similarity with the Arabidopsis AtAP1 protein, exhibiting 95.70% sequence identity (Figure 6).

### 2.7. Expression Analysis of IiAP1 and Nuclear Localization of the Encoding Product

**Figure 7:**
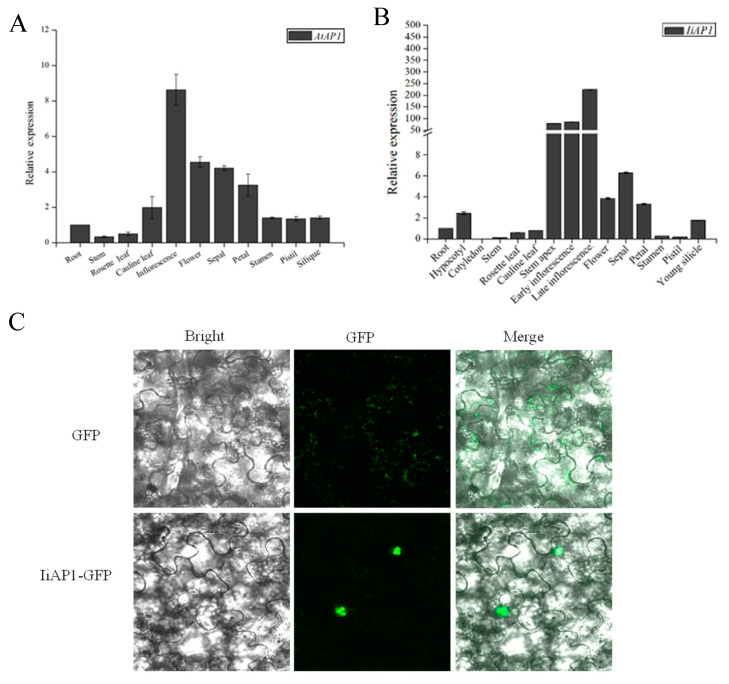
The expression patterns of *IiAP1* and subcellular localization of IiAP1 protein. (**A**): Expression patterns of *AtAP1* in different tissues and organs of Arabidopsis analyzed via quantitative RT-PCR, (**B**): Expression patterns of *IiAP1* in different tissues and organs of *I. indigotica* analyzed via quantitative RT-PCR. The error bars indicate the standard deviation. Statistical significance was assessed using Student’s *t*-test. (**C**): Subcellular localization of IiAP1 protein.

To examine the expression profiles of *IiAP1* in various tissues and organs of *I. indigotica*, quantitative real-time PCR (qRT-PCR) analysis was conducted. The reference gene used in this study was the actin gene of *I. indigotica* (GenBank accession No. AY870652.1). The normalization sample utilized for comparison was based on the root. The expression pattern is similar to the *AP1* of Arabidopsis (Figure 7A). *IiAP1* was not expressed in the vegetative stage and started to express in early inflorescence. In late inflorescence, the abundance of *IiAP1* mRNA reached its peak. Moreover, *IiAP1* was expressed in sepals and petals, but not as expressed in stamens and pistils. These results showed that *IiAP1* was involved in regulating the formation of floral meristems and the development of sepals and petals (Figure 7B). To gain deeper insights into the subcellular localization of IiAP1, we employed the transient expression of *IiAP1-GFP* in *Nicotiana benthamiana* leaf cells. The result revealed that the IiAP1-GFP fusion protein was localized to the nucleus (Figure 7C).

### 2.8. Ectopic Expression of *IiAP1* in Arabidopsis

**Figure 8:**
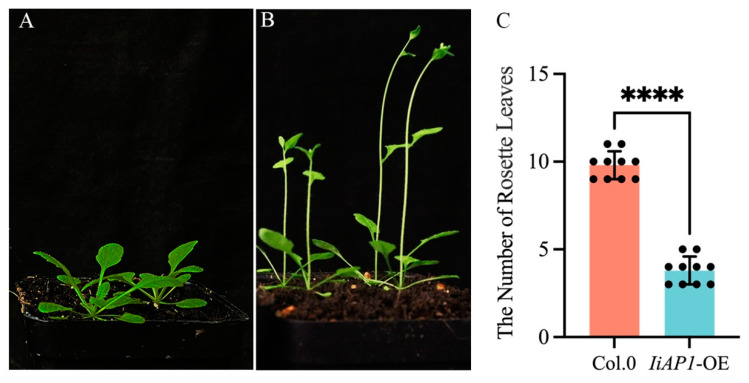
Early flowering phenotype of *IiAP1* ectopic expression Arabidopsis plants in T1 generation. (**A**): Wild-type Col-0 plant; (**B**): *IiAP1* ectopic expression Arabidopsis plant; (**C**): statistical analysis of the number of rosette leaves between *IiAP1* overexpressing Arabidopsis plants and Col-0 plants. Significant differences according to Student’s *t*-test are indicated, **** represents *p* < 0.0001.

**Figure 9:**
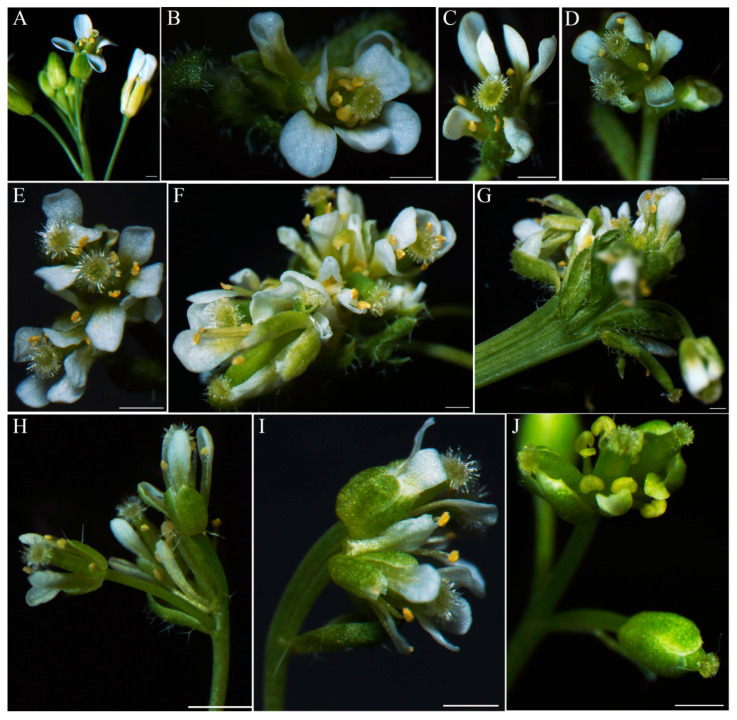
Phenotypic variations in the floral organs of *IiAP1* transgenic Arabidopsis plants. (**A**): Wild-type flower of Arabidopsis ecotype Col-0; (**B**–**J**): *IiAP1*-overexpressing Arabidopsis. (**B**,**C**): Unusual number of petals; (**D**): two pistils; (**E**,**F**): shoots transformed into terminal flowers; (**G**–**H**): inflorescence branches converted to solitary flowers; (**I**): petal–sepal mosaic structure; (**J**): deformed flowers.

Compared with the Arabidopsis wild type, the *IiAP1* ectopic expression Arabidopsis exhibited significantly advanced flowering times and a marked reduction in the number of rosette leaves at the bolting stage. The *IiAP1* ectopic expression lines developed three leaves before flowering, in contrast to the Col.0 lines, which produced approximately nine leaves (Figure 8A–C). The wild-type Arabidopsis flowers consisted of sepals (four), petals (four), stamens (six), and a pistil (one) (Figure 9A). However, the ectopic expression of *IiAP1* in Arabidopsis resulted in an abnormal flower phenotype, including abnormal inflorescence and flowers (Figure 9B–J). The ectopic expression of *IiAP1* resulted in an increase in the number of petals in Arabidopsis (Figure 9B,C). The aberrant flower was distinguished by the occurrence of two pistils, accompanied by an atypical quantity of petals and stamens encircling the pistils (Figure 9D). The primary shoots were transformed into compound terminal flowers, comprising three or more pistils surrounded by an abnormal number of sepals, petals, and stamens (Figure 9E,F). In addition, several *IiAP1* overexpressing lines exhibited abnormal inflorescence development, with lateral shoots developing into solitary and deformed flowers (Figure 9G,H,J). Furthermore, in a number of flowers in the *IiAP1* ectopic expression plant, the sepals were transformed into petal–sepal mosaic structures (Figure 9I).

## 3. Discussion

### 3.1. Identified 102 MADS-Box Genes from the Transcriptome of I. indigotica

MADS-box genes play regulatory roles in inflorescence development, flower induction, floral initiation, and morphogenesis, as well as fruit growth [3,4,5,6,41]. Previous studies identified 80 IiMADS genes in *I. indigotica* [42]. In this research, a total of 102 MADS-box genes were discovered in *I. indigotica* through the utilization of transcriptomics and genome data [40]. The gene counts are significantly lower than those in *Arabidopsis thaliana* (107 genes) [12], and maize (211 genes) [19], but they are higher than in rice (75 genes) [18]. According to their phylogenetic relationship, 102 IiMADS-box genes were divided into type II (MIKC^C^ and MIKC*) and type I (Mα, Mβ, and Mγ) subfamilies (Figure 1 and Figure 2). In addition, we investigated the conserved motifs of 102 protein sequences in *I. indigotica* (Figure 3) and identified that motif 1 was universally present in all IiMADS proteins, while motif 10 was exclusively present in the MIKC* subfamily and motif 9 was present solely in the MIKC^C^ subfamily. The variety of motifs within the IiMADS gene family indicates that these genes could have more potential functions. The exon–intron arrangement in type I genes is usually characterized by the absence of an intron or the presence of only one, whereas multiple introns are commonly observed in type II genes (Figure 4). It is noteworthy that protein motifs and exon–intron structures exhibit consistent conservation across diverse plant species [12,18,19]. Furthermore, there are 48 cis-regulatory elements located in the promoter regions of IiMADS genes (Figure 5A,B). The I-box (GATAA), which is a critical binding motif, plays a pivotal role among the light-responsive elements identified in plants [43]. The ABRE (PyACGTGGC) is crucial for plant responses to ABA, influencing their tolerance to osmotic and drought stresses [44,45]. The P-box (AA/CGACT) is responsive to GA and plays a crucial regulatory role in the transition of plant flowering [46]. Box 4 (ATTAAT) is very important for tissue-specific expression [47]. The analysis of *cis*-regulatory elements revealed that IiMADS genes are pivotal not only in the growth and development of *I. indigotica*, but also in regulating its tolerance.

The mechanisms of gene family formation include gene duplication, relocation, divergence, and coevolution [48]. Gene duplications are considered to be the result of various errors that occur during the replication and reconstruction processes of DNA [48] In this study, we found that there were seven chromosomes containing nine pairs of duplicated genes (Figure 5C). These findings demonstrated that the evolution of the MADS-box gene was primarily influenced by replication events. The Ka/Ks ratio was employed to analyze genome replication, and we calculated the Ka/Ks ratios of the IiMADS gene pairs. The orthologous MADS-box gene pairs exhibited a Ka/Ks < 1, indicating that the IiMADS genes experienced evolutionary pressure favoring conservation [49].

### 3.2. Type II MADS-Box Genes Belong to Well-Defined Subfamilies

As for the genes belonging to type II, the MIKC^c^ subtype is widely recognized for its exclusive presence in plants and plays a critical role in the development of floral organs. The phylogenetic study revealed that genes belonging to this subtype can be categorized into 12 subfamilies, including the following: AP1-like (A), AP3-like (B), AG-like (C/D), SEP-like (E), SVP-like, FLC-like, AGL6-like, AGL12-like, AGL17-like, TM3-like, TT16/PI, and MIKC* (Figure 2). Additionally, the *FLC*-like gene is present in both *I. indigotica* and *A. thaliana*. *FLC*-like genes play a crucial role in enabling plants to develop floral competence after extended exposure to cold temperatures. The *FLC* genes are known for their role in controlling plant flowering times by regulating vernalization and vernalization-independent pathways [50]. The absence of the FLC-like gene can be observed in plant species such as rice, cotton, and orchids, which do not exhibit a dependency on vernalization for their flowering process [19,51,52]. This suggests that the flowering of *I. indigotica* is dependent on undergoing vernalization. The *AGL6*-like gene *IiAGL6* negatively regulates flowering transition and plays an important role in stamen formation [30]. The *IiSVP* gene controls the size of floral organs [37]. *AGL17*-like genes play a role in the development of roots and the TM3 family represents an additional group of MADS-box genes that play a role in the autonomous pathway [21]. The phylogenetic analysis showed that 3 and 12 IiMADS genes were identified in the AGL17-like and TM3 families in *I. indigotica*, respectively.

Based on the ABCDE model, flower development and the determination of floral organ identity are attributed to the collective influence of multiple MADS-box genes [6]. In this study, we successfully identified genes that adhere to the ABCDE model, including AP1-like (three), AP3-like (three), AG-like (six), and SEP-like (four) genes (Figure 4). Our previous studies have elucidated the regulatory roles of *IiFUL*, *IiAP3*, *IiPI*, *IiSHP2*, and *IiSEP*-like genes in the flowering transition and floral organ development of *I. indigotica* [29,31,32,33,34,35,36]. The *IiFUL* gene regulates the silicles of *I. indigotica* indehiscent phenotype [31]. The AP3 and PI proteins play a regulatory role in the development of petals and stamens. The K domain of IiAP3 is responsible for specifying stamen identity, while the K domain of IiPI primarily contributes to petal formation. Additionally, the C-terminal region of IiPI is involved in representing stamen characteristics [29]. The *IiSHP2* gene is actively engaged in the developmental processes of silicles and seeds [36]. The *IiSEP*-like genes, including *IiSEP1*, *IiSEP2*, *IiSEP3*, and *IiSEP4*, govern the development of petals, sepals, stamens, and carpels [32,33,34,35].

### 3.3. Ectopic Expression of IiAP1 in Arabidopsis Promotes Early Flowering

The initiation of floral meristems started with the synergistic activation effect of *LFY* and *CAL* on *AP1* [53]. During the floral transition, LFY and a protein complex comprising FT and FD directly induce the activation of *AP1* expression [54]. The orthologs of Arabidopsis *AP1* in other plant species were isolated, and their functions in floral transition were investigated experimentally. We selected *IiAP1*, which belongs to the AP1-like sub-branch, for further functional study and found that the *IiAP1*-encoded protein is localized in the nucleus (Figure 7). The ectopic expression of *IiAP1* in Arabidopsis causes early flowering (Figure 8), indicating that similarly to *AP1* [55], *IiAP1* can promote the differentiation of floral meristems. Similarly, previous research has shown that transgenic Arabidopsis plants overexpressing *AP1* from various plant species including *Lilium longiflorum* [56], *Lactuca sativa* [57], *Fagopyrum esculentum* [58], and *Rosa chinensis* [59], exhibited an earlier flowering phenotype. Conversely, the down-regulation of *IiAP1* expression delayed flowering in *Rosa chinensis* [59], suggesting a conserved role of *AP1*-like genes in accelerating flowering, probably through promoting the early formation of floral meristems.

### 3.4. Ectopic Expression of IiAP1 in Arabidopsis Altered Flower Morphology

*AP1* is capable of determining the identity of Arabidopsis sepals and petals [60]. The flowers of the strong *ap1* single mutant contain bract-like sepals and lack petals, and the secondary flowers with the same structures can form in the axil of the first whorl organs, indicating that *AP1* is involved in the determination of floral meristem identity and the development of perianth [55]. AP1 activated *AP3* and *PI* gene expression to mediate the specification of petals [61]. In contrast, AP1 suppressed *AGL24* gene expression to prevent the reversion of floral meristems into inflorescence meristems [62]. The ectopic expression of an *AP1*-homologous gene from apples in Arabidopsis resulted in the abnormal development of floral organs and decreased fertility [63]. In *LMADS5,* an *AP1*-like gene from lilies, overexpressed Arabidopsis line sepals converted into carpel-like structures and petals transformed into stamen-like structures [56]. In the present study, the ectopic expression lines of *IiAP1* exhibited phenotypic traits such as the transformation of inflorescence branches into individual flowers, the development of terminal flowers, and the production of flowers with an increased number of petals, stamens, and pistils (Figure 9). Our findings suggest that *AP1*-like genes have the capacity to modify the identity of floral organs.

## 4. Materials and Methods

### 4.1. Plant Materials, RNA Extraction, and cDNA Synthesis

In the present study, *Isatis indigotica* Fortune (*I. indigotica* Fort.) and Arabidopsis ecotype Col-0 plants were used as materials. *I. indigotica* flowers were used to characterize the cDNA sequences. RNA was isolated from the floral organs of *I. indigotica* using the TaKaRa MiniBEST Plant RNA Extraction Kit (Takara, Code No. 9769) (Figure S3). First-strand cDNA was generated using the PrimeScript 1st Strand cDNA Synthesis Kit (Takara, Code No. 6215A). The full-length cDNA sequence of *IiAP1* was determined via 3′- and 5′-RACE methods using the SMARTer RACE cDNA Amplification Kit (Clontech, Code No. 634858).

### 4.2. Identification and Sequence Analysis of MADS Genes in I. indigotica

The transcriptomics dataset of *I. indigotica*, which was annotated (unpublished data from our group), and genome data [40] were used to identify the MADS-box gene family. The IiMADS gene family was identified using the reference sequence derived from the full-length transcriptome of *I. indigotica*, which was previously sequenced in our laboratory. The whole-genome protein sequences of other crops (*A. thaliana*, *Oryza sativa*, *Zea mays*, tomato, and wheat) were from the Plant JGI Database phytozome v13 (https://phytozome.jgi.doe.gov/pz/portal.html, accessed on 6 October 2022) [64]. First, all putative IiMADS proteins were identified using BLASTp (the e-value was set to 10^−5^) with the MADS protein sequence of Arabidopsis used as the reference [65]. Second, the PFAM protein family database (http://pfam.xfam.org/, accessed on 8 January 2021) was used to produce a Hidden Markov Model (HMM) file with the MADS and K domains (PF00319 and PF01486, respectively) [66,67]; then, an HMM model cutoff value of 0.01 was applied to compare the IiMADS protein sequences of *I. indigotica* in HMMER 3.0 [68]. Finally, NCBI-CDD (National Center for Biotechnology Information—Conserved Domain Database) (https://www.ncbi.nlm.nih.gov/Structure/bwrpsb/bwrpsb.cgi, accessed on 10 September 2021) was used to confirm the identity of IiMADS proteins containing the functional protein domains and to remove the missing structural domain sequences [69]. TBtools v2.142 software was used to identify the physical characteristics of IiMADS gene family members [70].

### 4.3. Phylogenetic Investigation and Categorization of the IiMADS Genes

The amino acid sequences of the MADS proteins from *I. indigotica*, Arabidopsis, rice, and maize were used for phylogenetic analysis. The ClustalW method with default parameters was utilized to conduct multiple sequence alignments for all chosen MADS sequences. Based on MEGA 7.0 software, the phylogenetic trees were constructed using the Neighbor-Joining (NJ) method [71]. Furthermore, the phylogenetic tree underwent visual enhancement through the use of EvolView (https://www.evolgenius.info//evolview/#login, accessed on 26 May 2020) [72].

### 4.4. Exploration of Gene Structure, Conserved Motifs, and Cis-Acting Elements in the IiMADS Genes

The positional information of Type I and Type II IiMADS genes was extracted from the genome annotation files of *I. indigotica* using TBtools v2.142 software, and the exon–intron gene structure was illustrated using the online software GSDS (https://gsds.gao-lab.org/index.php, accessed on 10 December 2014). For the purpose of discovering conserved motifs within the IiMADS proteins, we used Multiple Em for Motif Elicitation (https://meme-suite.org/meme/tools/meme, accessed on 17 December 2022) [73]. The motifs that were shared among the genes belonging to one of the three similarity groups were designated as group-specific signatures. TBtools v2.142 [72] was used to visualize protein structures. The gene location information on chromosomes was extracted from the GFF annotation files of the MADS-box family. Based on this location information, we extracted the promoter sequences of the first 2 kb upstream of the genes. The completed sequence information files were uploaded to the PlantCARE (http://bioinformatics.psb.ugent.be/webtools/plantcare/html/, accessed on 1 January 2002) online website for analysis. Then, the analysis results were filtered, and finally TBtools v2.142 software was used to visualize the filtered results.

### 4.5. Chromosomal Location and Collinearity Analysis of the IiMADS Genes

TBtools v2.142 software was utilized to extract the positional information of transcripts corresponding to the genes in the genome and their respective protein sequences. The protein sequences were then subjected to BLAST alignment. MCScanX (https://github.com/wyp1125/MCScanX, accessed on 4 January 2012) was employed to identify collinearity, and TBtools v2.142 was used to generate and beautify images based on the positional information.

### 4.6. Protein–protein Interaction Network of IiMADS Genes

The protein sequence of the MADS gene family from *I. indigotica* was uploaded to the STRING database (https://cn.string-db.org/, accessed on 6 January 2023) for analysis, using *Arabidopsis* as a reference. The Cytoscape v3.7.0 software [74] was then utilized to visualize, beautify, and further analyze the results, ultimately yielding a protein–protein interaction network diagram.

### 4.7. Analyses of the IiAP1 Expression Patterns Using qRT-PCR

Total RNA was isolated from various tissues and floral organs of *I. indigotica* and Arabidopsis using the TaKaRa MiniBEST Plant RNA Extraction Kit (Takara, Code No. 9769), respectively (Figure S3). And the cDNA was synthesized using the PrimeScript 1st Strand cDNA Synthesis Kit (Takara, Code No. 6215A). qRT-PCR (quantitative real-time PCR) was adopted to analyze the expression patterns of *IiAP1*, and qRT-PCR primers were presented in Table S2 (Figure S4). The relative expression level was determined via the 2^−ΔΔCt^ method [75] and the root was utilized as a standardized sample. The reference gene was *I. indigotica Actin* (AY870652.1). The dataset comprised three biological replicates, each consisting of three technical replicates, which underwent statistical analysis using Student’s *t*-test.

### 4.8. Subcellular Localization of IiAP1 Protein

Two-week-old *Nicotiana benthamiana* leaf cells were transformed using *Agrobacterium tumefaciae* containing plasmid pCAMBIA1302-*IiAP1* [34]. Green fluorescence was observed under a laser scanning confocal microscope with 488nm excitation after 60 h.

### 4.9. Obtaining Transgenic Arabidopsis Plants Ectopic Expression of IiAP1

In order to study the regulatory function of *IiAP1*, wild-type Col-0 Arabidopsis plants were transformed via the *Agrobacterium*-mediated floral dip method. GV3101 strains carrying recombinant plasmids pCAMBIA1302-*IiAP1* were cultured in an LB liquid medium containing 0.05 mg/mL Kan, 0.05 mg/mL Gent, and 0.05 mg/mL Rif to OD_600_ = 0.8. The bacterial cells were resuspended with infiltration solution (1/2MS_0_, 50 g/L sucrose, 0.5 g/L MES, pH = 5.7) per liter containing 50 μL of 1 mg/mL 6-BA. Siliques on Col-0 Arabidopsis plants were cut off and the aerial parts were immersed in a suspension liquid of the GV3101 cells for 30 s. The treated plants were maintained in the dark for 24 h at 100% humidity. Afterward, the plants were cultured at 23 ± 2 °C under a long-day photoperiod. Seeds were screened on an MS_0_ medium containing 0.025 mg/mL hygromycin. Transgenic plants with 3–4 true leaves growing in a mixed matrix of vermiculite, perlite, and soil (2:1:1) were identified via PCR. Transgenic phenotypes were confirmed in the T1 and T2 generations.

## 5. Conclusions

In this research study, we identified a total of 102 IiMADS-box genes, which were subsequently categorized into two distinct groups (type I and type II), and they were further classified into Mα (22), Mβ (17), Mγ (11), MIKC^c^ (47), and MIKC* (5) according to their phylogenetic relationships. Type I and type II IiMADS genes exhibited notable differences in their structural characteristics and conserved motifs. At least 18 *IiMADSs* belong to the well-known classic ‘ABCDE’ model genes in *I. indigotica*, indicating their potential roles in the floral organogenesis of *I. indigotica.* Furthermore, we selected *IiAP1* for further functional study. Our data showed that ectopic expression of *IiAP1* in Arabidopsis not only promotes flowering, but also affects inflorescence development and floral organ identity. The results of this study provide references for an in-depth understanding of the biological function of the *I. indigotica* MADS-box genes and the mechanism of *I. indigotica* flower development.

## Figures and Tables

**Table 1:** Duplication pairs for MADS-box genes in *I. indigotica*.

Multiple-Copy Gene Pairs	Synonymous Substitution Rate (Ka)	Nonsynonymous Substitution Rate (Ks)	Ka/Ks
IiMADS91/IiMADS69	0.0921623	0.502525	0.183398
IiMADS36/IiMADS38	0.0265616	0.401908	0.0660888
IiMADS90/ IiMADS42	0.0194865	0.387274	0.050317
IiMADS40/ IiMADS89	0.0672956	0.511771	0.131496
IiMADS64/ IiMADS63	0.037241	0.266845	0.139561
IiMADS20/ IiMADS21	0.0850036	0.452594	0.187814
IiMADS45/ IiMADS47	0.0603465	0.288191	0.209397
IiMADS30/ IiMADS96	0.0776146	0.39195	0.198022
IiMADS5/ IiMADS6	0.0439878	0.195654	0.224824

## Data Availability

The original contributions presented in the study are included in the article/Supplementary Materials; further inquiries can be directed to the corresponding authors.

## Supplementary Materials

The following supporting information can be downloaded at https://www.mdpi.com/article/10.3390/plants14010129/s1.
