# Supplementary material for: Characterization of MADS-Box Gene Family in Isatis indigotica and Functional Study of IiAP1 in Regulating Floral Transition and Formation
Source: Plants (Basel). 2025 Jan 4;14(1):129. doi: 10.3390/plants14010129 (PMC11723362; doi:10.3390/plants14010129)
Supplement: Supplementary file 1 [file plants-14-00129-s001.zip › plants-3291080-supplementary.pdf]

Characterization of MADS-Box Gene Family in *Isatis indigotica* and Functional Study of *IiAP1* in Regulating Floral Transition and Formation

Yanqin Ma [1,2,3,4†], Yanhong Lan [1,2,3†], Ju Li [1,2,3], Haicheng Long [1,2,3], Yujie Zhou [1,2,3], Zhi Li [1,2,3], Mingjun Miao [1,2,3], Jian Zhong [1,2,3], Haie Wang [1,2,3], Wei Chang [5], Ziqin Xu [4] and Liang Yang [1,2,3*]

[1] Horticulture Research Institute, Sichuan Academy of Agricultural Sciences, Chengdu 610066, Sichuan, People's Republic of China; dora0514@sina.cn (Y.M.); yanhong_lan2022@163.com (Y.L.); dandelionlj@126.com (J.L.); longhaicheng2024@126.com (H.L.); 15680897967@sina.cn (Y.Z.); lz20031977@126.com (Z.L.); 7200175@uestc.edu.cn (M.M.); zhongjian15@scsaas.cn (J.Z.); hewang-scnky@scsaas.cn (H.W.)

[2] Key Laboratory of Horticultural Crops Biology and Germplasm Enhancement in Southwest Regions, Ministry of Agriculture and Rural Affairs of the P.R. China, Chengdu 610066, Sichuan, People's Republic of China;

[3] Vegetable Germplasm Innovation and Variety Improvement Key Laboratory of Sichuan Province, Chengdu 610066, Sichuan, People's Republic of China;

[4] Key Laboratory of Resource Biology and Biotechnology in Western China (Ministry of Education) Provincial Key Laboratory of Biotechnology, College of Life Sciences Northwest University, Xi'an 710069, Shannxi, People's Republic of China; ziqinxu@nwu.edu.cn

[5] Sichuan Institute of Edible Fungi, Chengdu 610066, Sichuan, People's Republic of China; changwei972@126.com

* Correspondence: yangliang_saas@foxmail.com

† These authors have contributed equally to this work.

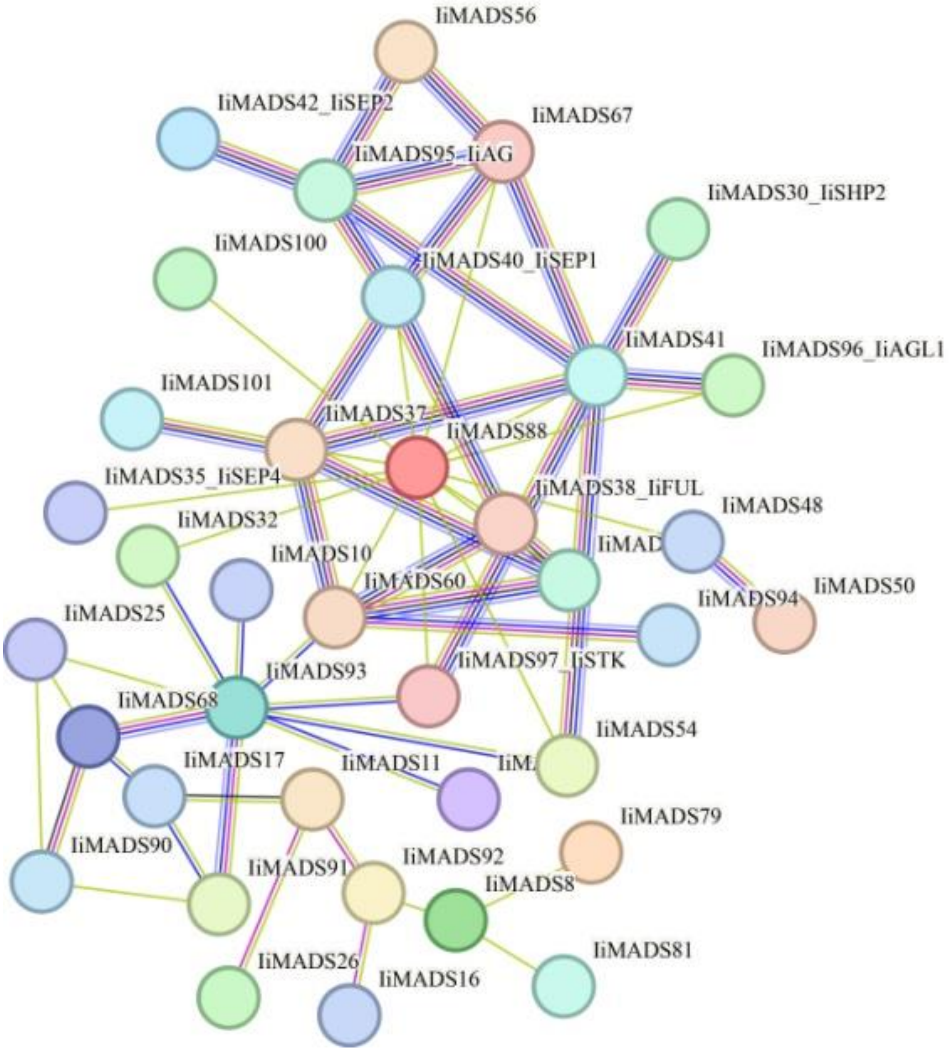

Figure S1. The protein interaction network of the *Isatis tinctoria* MADS-box gene family.

```
AAACACAAGTCTTATTATAAAGGAAAGAAAGAAAAACTTTCCTAATTGGATCATACCAAAGCCTGGGCTCTTC
TTTATCTCTCTTCTTGTAGTTTCTTGATTTGTTTGGTTCTTTTAGAGGAAATAGTTTCTTAAAAAAGGATCAAAA
atg gga agg ggt agg gtt cag ttg aag agg ata gag aac aag atc aat aga cag gtg aca
 M   G   R   G   R   V   Q   L   K   R   I   E   N   K   I   N   R   Q   V   T
ttc tcg aaa aga aga gct ggt ctt ttg aag aaa gct cat gag att tct gtt ctc tgt gat
 F   S   K   R   R   A   G   L   L   K   K   A   H   E   I   S   V   L   C   D
gct gaa gtt gcc ctt gtt gtc ttc tcc cat aag ggg aaa ctc ttc gaa tac tcc act gat
 A   E   V   A   L   V   V   F   S   H   K   G   K   L   F   E   Y   S   T   D
tct tgt atg gag aag ata ctt gaa cgc tat gag agg tac tct tac gcc gag aga cag ctt
 S   C   M   E   K   I   L   E   R   Y   E   R   Y   S   Y   A   E   R   Q   L
att gca cct gag tcc gac gtc aat acg aac tgg tcg atg gag tat aac agg ctt aag gct
 I   A   P   E   S   D   V   N   T   N   W   S   M   E   Y   N   R   L   K   A
aag att gag ctt ttg gag aga aac cag agg cac tat ctt ggg gaa gac ttg caa gca atg
 K   I   E   L   L   E   R   N   Q   R   H   Y   L   G   E   D   L   Q   A   M
agc cct aag gag ctc cag aat ctg gag cag cag ctt gac act gct ctt aag cat atc cgc
 S   P   K   E   L   Q   N   L   E   Q   Q   L   D   T   A   L   K   H   I   R
tct agg aaa aac caa ctg atg tac gac tcc atc aat gag ctc caa aga aag gag aaa gcc
 S   R   K   N   Q   L   M   Y   D   S   I   N   E   L   Q   R   K   E   K   A
ata cag gaa caa aac agc atg ctt tcc aaa cag atc aag gag agg gaa aag gtt ctt agg
 I   Q   E   Q   N   S   M   L   S   K   Q   I   K   E   R   E   K   V   L   R
gca caa caa gag caa tgg gac cag cag aac cat ggc caa aac atg cct ccg cct ccg ccc
 A   Q   Q   E   Q   W   D   Q   Q   N   H   G   Q   N   M   P   P   P   P   P
ccg cag cag cat caa atg cag cat cca tac atg ctc tct cat cag cca tct cct ttt ctc
 P   Q   Q   H   Q   M   Q   H   P   Y   M   L   S   H   Q   P   S   P   F   L
aac atg ggt ggc ctg tat caa gaa gaa gat cca atg gca atg agg agg aac gac ctt gat
 N   M   G   G   L   Y   Q   E   E   D   P   M   A   M   R   R   N   D   L   D
ctg tcc ctt gaa ccc gtc tac aac tgc aac ctt ggc tgc ttt gcc gca tga AGATCCATATA
 L   S   L   E   P   V   Y   N   C   N   L   G   C   F   A   A   -
TATTTTTATAATCATCCATAATAAAAAACATTTAGCCACATTATATTTTGGCTAGCCCCTTTTTATATCCAATTA
GTTAAATATATTTTGGCAAGTTCCATGTTCCTATGTAATATAAATTTAGCAAGTTCTTTCTTTTCTCTTTATATTT
TTTTAAACTGTTTTTGCTTCCGTATGTGGCAAAATTATATTGTAATATATTGATATTCTGTTTTAGTTTGGAATTA
TTGGCATATAAAATTTTTATCGAAATTATATTGGAATTTTAAAAAAAAAAAAAAAAAAAAAAAAA
```

Figure S2. The cDNA sequence of *IiAP1* and the amino acid sequence of the encoded product. The sizes of the full-length cDNA and the coding sequence of *IiAP1* are 1002 bp and 771 bp, respectively. IiAP1 is constituted by 256 amino acids.

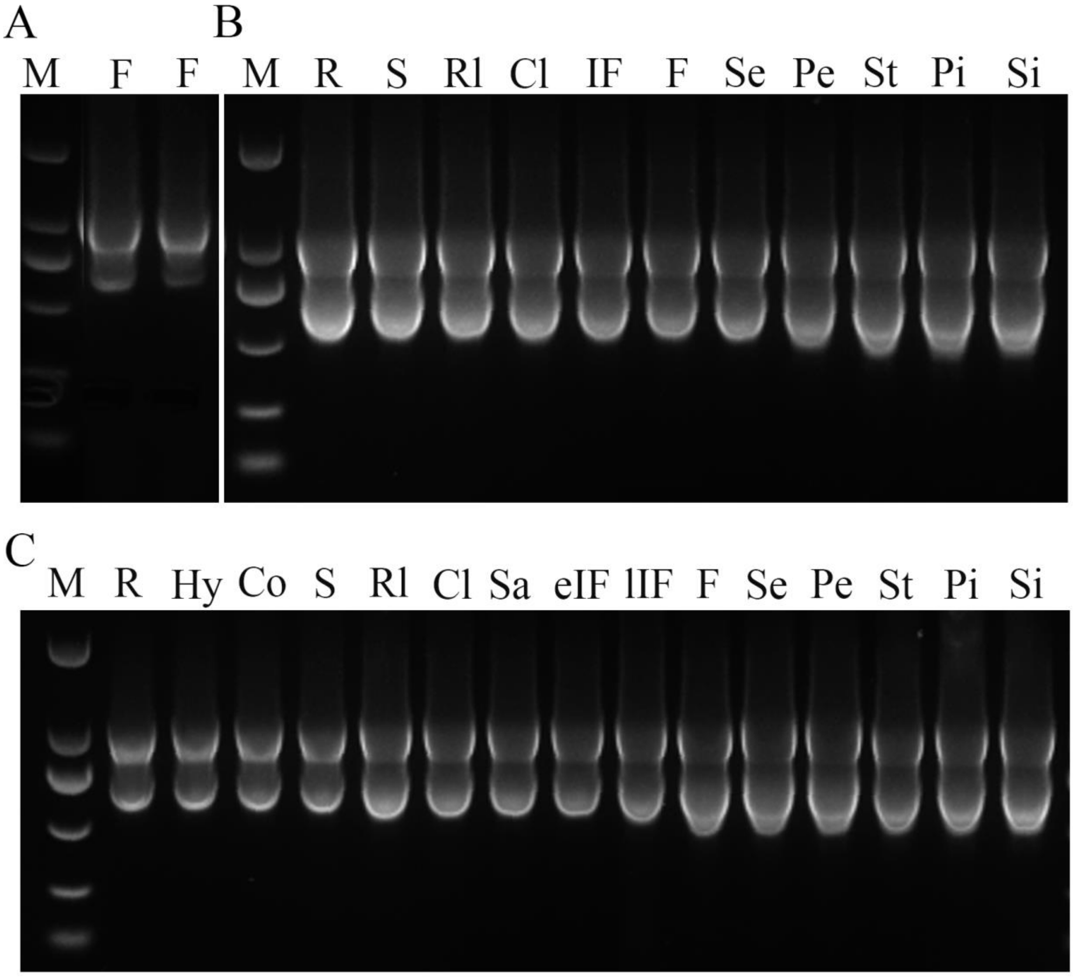

Figure S3. The total RNA was isolated from various tissues and organs of *Arabidopsis thaliana* and *Isatis indigotica*. A: the RNA was isolated from the floral organs of *I. indigotica*; B: the RNA was extracted from different tissues and floral organs of *Arabidopsis thaliana*; C: the RNA was extracted from different tissues and floral organs of *I. indigotica*. M: DL2000 DNA marker; R: Root; Hy: Hypocotyl; Co: Cotyledon; S: Stem; Rl: Rosette leaf; Cl: Cauline leaf; Sa: Stem apex; IF: inflorescence; eIF: early inflorescence; lIF: Late inflorescence; F: Flower; Se: Sepal; Pe: Petal; St: Stamen; Pi: Pistil; Si: silique or young silicle.

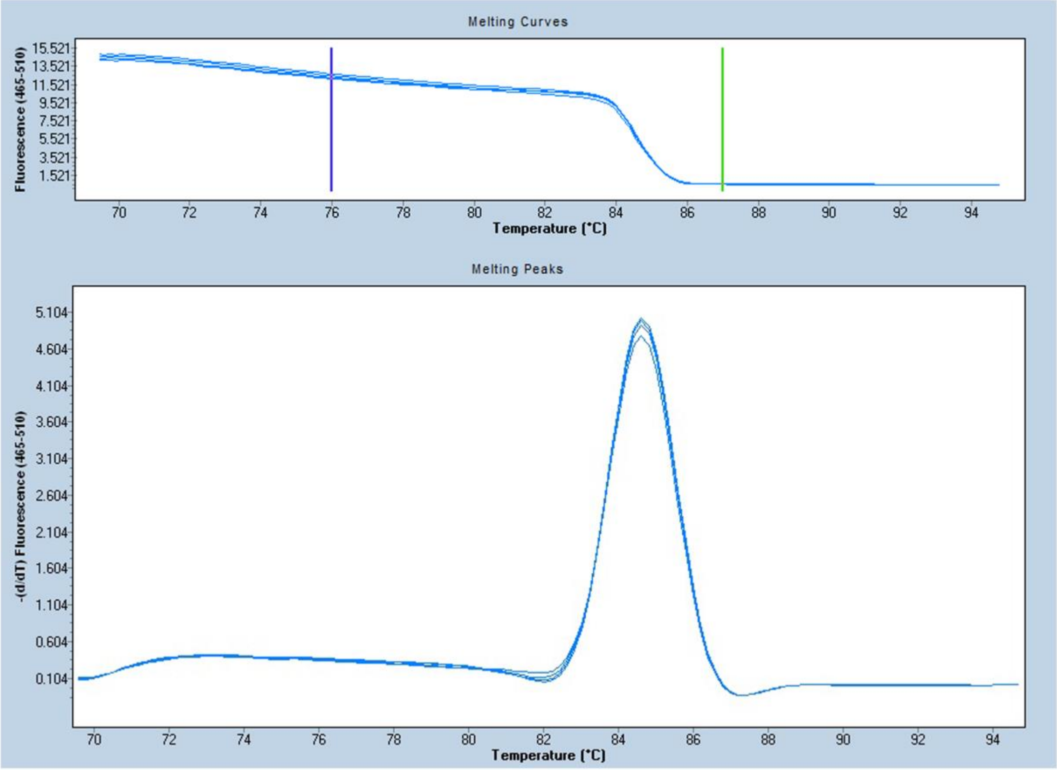

Figure S4. The melting curves of primer for qRT-PCR.

Table S1. Physicochemical properties of IiMADS gene family members. MW: Molecular Weight, pI: Isoelectric Point, TMHMM: Transmembrane helices, Loc: Subcellular Localization, GRAVY: Grand average of hydropathicity.

Gene Name	Length (aa)	CDS sequence (bp)	MW(Da)	pI	Loc	TMHMM	Instability Index	Aliphatic Index	GRAVY
IiMADS1	220	660	24609.78	6.84	nucleus	NO	47.24	78.86	-0.544
IiMADS2	247	741	27419.53	4.69	nucleus	NO	48.61	76.6	-0.43
IiMADS3	306	918	34384.83	4.36	nucleus	NO	50.62	73.95	-0.7
IiMADS4	322	966	36162.85	4.4	nucleus	NO	48.28	75.12	-0.659
IiMADS5	183	549	20687.1	6.98	nucleus	NO	53.09	80.44	-0.614
IiMADS6	183	549	20645.16	8.91	nucleus	NO	47.42	79.95	-0.607
IiMADS7	112	336	12055.04	10.15	nucleus	NO	56.54	77.5	-0.237
IiMADS8	208	624	23054.35	4.06	nucleus	NO	39.34	81.11	-0.42
IiMADS9	157	471	18248.73	8.41	nucleus	NO	48.8	60.32	-1.073
IiMADS10	216	648	24652.13	8.37	nucleus	NO	55.52	75.88	-0.644
IiMADS11	379	1137	43362.44	8.66	nucleus	NO	40.91	55.78	-0.855
IiMADS12	1113	3339	125344.96	8.63	nucleus	NO	48.8	57.64	-0.911
IiMADS13	337	1011	38665.48	7.98	nucleus	NO	59.46	63.09	-0.771
IiMADS14	269	807	30354.1	6.75	nucleus	NO	41.06	73.57	-0.686
IiMADS15	264	792	29799.48	6.51	nucleus	NO	47.99	74.96	-0.647
IiMADS16	312	936	35552.11	9	nucleus	NO	39.79	73.14	-0.707
IiMADS17	224	672	25178.31	5.4	nucleus	NO	41.02	67.1	-0.645
IiMADS18	241	723	27322.85	8.87	nucleus	NO	33.33	74.07	-0.631
IiMADS19	308	924	34832.1	7.77	nucleus	NO	38.74	75.68	-0.592
IiMADS20	308	924	34808.03	7.15	nucleus	NO	39.41	74.42	-0.612
IiMADS21	305	915	34542.58	7.23	nucleus	NO	39.3	72.59	-0.671
IiMADS22	193	579	22063.88	9.61	nucleus	NO	31.38	78.29	-0.754
IiMADS23	260	780	29319.96	9.27	nucleus	NO	46.59	67.81	-0.696
IiMADS24	189	567	21587.76	9.38	nucleus	NO	42.34	74.87	-0.515
IiMADS25	260	780	29249.86	6.25	nucleus	NO	44.34	70.88	-0.582
IiMADS26	248	744	27623.78	4.95	nucleus	NO	62.6	63.35	-0.712
IiMADS27	283	849	32166.6	8.48	nucleus	NO	37.02	64.06	-0.501
IiMADS28	283	849	32119.53	7.99	nucleus	NO	39.65	65.44	-0.478
IiMADS29	239	717	27109.07	8.93	nucleus	NO	39.07	58.74	-0.495
IiMADS30_IiSHP2	246	738	28134.69	9.23	nucleus	NO	63.43	82.8	-0.827
IiMADS31	247	741	28192.79	9.16	nucleus	NO	54.91	79.31	-0.807
IiMADS32	253	759	29040.82	8.51	nucleus	NO	41.14	73.16	-0.66
IiMADS33_IiSEP3-2	236	708	27189.74	7.01	nucleus	NO	50.96	80.59	-0.74
IiMADS34	258	774	28829.25	6.2	nucleus	NO	58.76	79.77	-0.808
IiMADS35	261	783	29664.56	9.3	nucleus	NO	54.27	72.91	-0.757

_IiSEP4									
IiMADS36 _IiAP1	256	768	30156.37	8.56	nucleus	NO	66	73.55	-0.927
IiMADS37	187	561	22199.63	9.43	nucleus	NO	60.03	87.59	-0.691
IiMADS38 _IiFUL	241	723	27532.46	9.44	nucleus	NO	54.55	85.77	-0.683
IiMADS39 _IiSEP3	250	750	28932.8	8.28	nucleus	NO	51.67	80.76	-0.766
IiMADS40 _IiSEP1	251	753	28709.59	8.49	nucleus	NO	40.9	80.44	-0.739
IiMADS41	173	519	20203.34	9.39	nucleus	NO	48.03	96.94	-0.423
IiMADS42 _IiSEP2	250	750	28563.5	8.65	nucleus	NO	41.07	81.88	-0.692
IiMADS43	213	639	24359.79	9.07	nucleus	NO	55.65	73.29	-0.843
IiMADS44	240	720	27543.7	9.35	nucleus	NO	51.36	76.42	-0.758
IiMADS45	268	804	30767.37	9.37	nucleus	NO	52.02	78.96	-0.706
IiMADS46	233	699	26817.8	9.5	nucleus	NO	48.86	93.3	-0.718
IiMADS47	217	651	25012.6	9.37	nucleus	NO	54.42	81.8	-0.866
IiMADS48	211	633	23977.72	7.67	nucleus	NO	67.64	85.97	-0.427
IiMADS49	290	870	33465.74	9.44	nucleus	NO	58.83	74	-0.813
IiMADS50	240	720	27562.37	8.52	nucleus	NO	46.38	86.92	-0.712
IiMADS51	209	627	24702.43	9.35	nucleus	NO	49.54	88.13	-0.79
IiMADS52	182	546	21234.45	9.07	nucleus	NO	62.52	93.24	-0.612
IiMADS53	226	678	25666.93	6.93	nucleus	NO	57.18	82.88	-0.793
IiMADS54	260	780	30785.77	6.39	nucleus	NO	69.15	78.38	-1.04
IiMADS55	180	540	20595.67	9.34	nucleus	NO	48.37	95.33	-0.624
IiMADS56	208	624	24085.92	9.18	nucleus	NO	49.23	88.61	-0.627
IiMADS57	202	606	23122.76	9.35	nucleus	NO	55.73	82.48	-0.589
IiMADS58	264	792	29532.51	8.95	nucleus	NO	49.34	90.49	-0.636
IiMADS59	196	588	21901.95	6.02	nucleus	NO	42.6	93.57	-0.43
IiMADS60	215	645	24416.82	8.37	nucleus	NO	52.08	86.65	-0.622
IiMADS61	199	597	23489.26	9.59	nucleus	NO	54.57	87.64	-0.51
IiMADS62	218	654	25089.1	9.21	nucleus	NO	52.75	93.35	-0.489
IiMADS63	219	657	25313.34	8.81	nucleus	NO	61.15	83.24	-0.584
IiMADS64	219	657	25524.23	7.62	nucleus	NO	57.2	85.39	-0.74
IiMADS65	155	465	17950.91	9.46	nucleus	NO	50.19	94.84	-0.308
IiMADS66	232	696	27148.63	7.84	nucleus	NO	45.88	83.23	-0.835
IiMADS67	174	522	20564.7	9.51	nucleus	NO	40.53	89.02	-0.595
IiMADS68	271	813	31477.76	9.58	nucleus	NO	50.45	77.23	-0.746
IiMADS69	163	489	18624.78	9.61	nucleus	NO	48.84	69.45	-0.548
IiMADS70	209	627	23536.18	9.21	nucleus	NO	46.55	70.38	-0.568
IiMADS71	165	495	18659.6	9.81	nucleus	NO	59.17	70.91	-0.588
IiMADS72	184	552	21289.14	9.14	nucleus	NO	60.08	66.25	-1.004

IiMADS73	80	240	9473.15	10.98	nucleus	NO	55.65	87.75	-0.703
IiMADS74	285	855	32939.27	8.64	nucleus	NO	52.49	62.95	-0.86
IiMADS75	338	1014	38698.36	6.01	nucleus	NO	54.01	63.22	-0.771
IiMADS76	167	501	18826.52	9.26	nucleus	NO	45.67	66.59	-0.721
IiMADS77	237	711	27426.46	7.13	nucleus	NO	48.99	73.67	-0.737
IiMADS78	269	807	30343.15	5.98	nucleus	NO	50.41	64.2	-0.577
IiMADS79	285	855	32892.29	8.84	nucleus	NO	48.79	61.58	-0.851
IiMADS80	271	813	30846.29	4.62	nucleus	NO	41.7	71.14	-0.626
IiMADS81	189	567	21782.06	9.57	nucleus	NO	65.12	71.22	-0.75
IiMADS82	238	714	27226.37	9.08	nucleus	NO	52.68	86.01	-0.508
IiMADS83	239	717	27521.71	9.41	nucleus	NO	49.64	87.28	-0.456
IiMADS84	375	1125	43090.49	5.98	nucleus	NO	55.74	67.6	-0.785
IiMADS85	382	1146	43885.41	5.98	nucleus	NO	55.21	67.12	-0.763
IiMADS86	343	1029	39264.73	6.41	nucleus	NO	40.77	86.97	-0.502
IiMADS87	154	462	16856.23	9.1	nucleus	NO	46.26	90.65	-0.251
IiMADS88	235	705	26289.56	5.18	nucleus	NO	50.26	82.13	-0.414
IiMADS89	265	795	30425.56	8.7	nucleus	NO	49.88	66.68	-0.715
IiMADS90	369	1107	42350.33	5.81	nucleus	NO	55.45	63.2	-0.799
IiMADS91	176	528	19714.68	9.75	nucleus	NO	59.34	67.61	-0.646
IiMADS92	171	513	20074.74	9.95	nucleus	NO	47.46	88.36	-0.603
IiMADS93	185	555	21009.27	8.94	nucleus	NO	48.58	84.86	-0.426
IiMADS94	219	657	25172.48	7.76	nucleus	NO	53.66	75.71	-0.844
IiMADS95 _IiAG	287	861	32985.26	9.59	nucleus	NO	57.54	73.76	-0.782
IiMADS96 _IiAGL1	173	519	19816.21	8.84	nucleus	NO	58.32	77.75	-0.907
IiMADS97 _IiSTK	231	693	26376.83	9.36	nucleus	NO	46.62	83.2	-0.691
IiMADS98 _IiSVP	241	723	27248.49	5.61	nucleus	NO	58.04	80.54	-0.807
IiMADS99	283	849	31763.74	7.63		NO	57.74	80.64	-0.719
IiMADS100	156	468	17742.55	9.54	nucleus	NO	49.85	100	-0.557
IiMADS101	150	450	16982.47	9.65	nucleus	NO	61.78	83.87	-0.599
IiMADS102	296	888	33795.51	9.42	nucleus	NO	45.9	90.61	-0.647

Table S2. Primers used for RACE experiments, construction of vector and qRT-PCR.

Name	Sequence (5′→3′)	Note
3′-GSPIiAP1	GACATTCTCGAAAAGAAGAG	3′-RACE
5′-GSPIiAP1	GGTGCAATAAGCTGTCTCTCGGCGTAAG	5′-RACE
IactinF	TATTGTTGGTCGTCCCAGGC	*IiActin*
IactinR	ACGACCACTGGCGTAAAGAG	*IiActin*
IiAP1-OE-F	GCGCATATGATGGGAAGGGGTAGGGTTCAG	Construction of vector
IiAP1-OE-R	GCGGAATTCTCATGCGGCAAAGCAGCCAAG	Construction of vector
IiAP1-QRT-F	CCCGCAGCAGCATCAAAT	qRT-PCR
IiAP1-QRT-R	TCAAGGTCGTTCCTCCTCATT	qRT-PCR

File S1. The amino acid sequences of IiMADS proteins from *I. indigotica*

>IiMADS1
MGGLKKKIPTDKMIEKKDSKSVAFSKRRKGLYSKASQLCLLSRAQVAILVTPPCSKSY
VSFYSFGHSSVDSVVSSFLANQRPREDLGSEFWWQDESLAELEDPNELSEALDSISRT
LQNLKGLRSAAMDMKKKEVVVYGTHHQQLDQSQTLNLQSTCASICIREDPPENFQR
LVKKTKEVDQIVAVSDKDAVDRNQTLDFQSSSASLRIQDVSPENFEGA*
>IiMADS2
MVKRGGTKRKIEMKKITSAASASVTLSKRRSGLYSKVSELCLLTDAQVAVLSTPLSSN
SYCNFFSFGHTSVDSIVTAFLNGERPVRVEAATKRESDDIGICFARHQLGLPLWCDDDR
LVRSNNLEELTDAINSVSRMLANIEELRSGDALKHQAFANDEDNEPLMNNNETTDQT
LVMKEQDQIMAICDSFSVPDKNNNSSFCVPENHDNNNNALCLYDESHESDPDFDMTF
TLSELYAISDSTPPCV*
>IiMADS3
MVKGTKRKIEIKKIANRTSVATSFTKRSHGLHSKASELCLLTGAQIAVLATPSSSKSNVS
FFSFGHSSVEAVVSAFLAGKSPPPVPEETKETREDLGICMARKNLGLGFWWDDEERF
KSREDIMEAIESMKILLSDVKALRSDLHRQNPNEAFLEKNKESDDGTADQTLVLDDTT
EHDQILSTSDSLSRSNNKKANLDEWSDMDLDQLLRDFDDDVPDEEEPIVSFSEETRNS
NNNNTNLDEWSDMDLDQLLKDFDDDLPDEEEPIVPFSEETWNSNNNALLSPAAGNLE
EDMGLDFEKICEEFPV*
>IiMADS4
MVKGTKRKIEIKKIANRTSVATSFTKRSHGLHSKASELCLLTGAQIAVLATPSSSKSNVS
FFSFGHSSVEAVVSAFLAGKSPPPVPEETKETREDLGICMARKNLGLGFWWDDEERF
KSREDIMEAIESMKILLSDVKALRSDLHRQNPNEAFLEKNKESDDGTADQTLVLDDTT
EHDQILSTSDSLSRSNNKKANLDEWSDMDLDQLLRDFDDDVPDEEEPIVSFSEETRNS
NNNNTNLDEWSDMDLDQLLKDFDDDLPDEEEPIVPFSEETWNSNNNALLSPAAGNLE
EDMGLDFEKICEDPFANVSHFSVMTLHPNEIL*
>IiMADS5
MGGTKRKIKIEKIEKKTVRSVAFTKRRNGLFRKASELCLLSSSTQIAILATPPSSNSHAS
FYSFGHSSVDNVVFSLLNGRSPLPVDQDYKSGLGFWWEDHERFDRLENIDELRKATD
AISRMLNNLRLRLDAVQSNQRDHGGLVIHQEEFLQLENTNTNNNEEISNRFEGASGSG
SLVENEVY*
>IiMADS6
MGGTKRKIKIEKIKKKTAKSVAFSKRRNGLFRKASELCLLFSSTQIAILATPPSSNSHAS
FYSFGHSSVDNVVSSLLNGRSPLPIDQYNKSGLGFWWEDHERFDRLENIDELRKATD
AISRMLNNLRLRLDAVQSNQRDHGGLVIHQEEFLQLENTNTNNNEEISNRFEGASGCG
SLVENEVY*
>IiMADS7
MVKGGTKRKIEIKKIENRLSMATSFTKRSNGLHSKASELCLLTGAQIVVLATPSSSKSN
MSFFSFGHSSVEAVVSAFLARKSPPPLPVETKETREDLGICMARKNLGSSSKP*
>IiMADS8
MATSFTKRSNGLHSKASELCLLTGAQIAVLATPSSSKSNVSFFSFGHSSVEAVVSAFLA
GKSPPPLPVETKETREDLGICMARKNLGLGFWWDDDERFKSLEDIMEAIESVKSLLTD
ILSTSDSLSNNNNNNANLDEWSDMDLDQLLNDSDDELPENQTGEEDHQIVSLPEKNC

NNVEEDMDLDFETIFEGLYSDDETNEFVQSLLMI*
>IiMADS9
MDARKTKMEKVQARNKKHVTFSRRRSALFKKASELAMTCNAEVGIVIFSPGDKPYS
FGKPSLDVISERFKNENEESENIGEGSIAELEKLHKHLNSINEKIDAEKKRGKEYQDWL
ESAGEKRFEKSLMELTHDELKENKAKQEKMHDKQDPCFEFKL*
>IiMADS10
MEDEEASSSTCLLLTEQKPLQIPKVLVNQKEETKLQNPKTTKGRQKIEIKRIKKESQRQ
VTFSKRRSGLFKKAAELSVLSGAQIGIITFSRRGRIYSFGNVDTLIQKYLRKSPVMLRT
HHGGDVANEEEDADGLKWWERAVESVPEEEMEEYMTALSGLRDNLWTRICRLGGD
RTVQMNAPAFLNPMAAIDWKMTDETVRNDQGQAGPCYAWELAF*
>IiMADS11
MVGGPRQNASVMDPNTTMASSSSFFTSSSTPMTKKTNKSRQVRPTLFQKQSSLKTKS
LTKKQATVFKKAYELSTLCGIEVCVIYYGSDGELKTWPEDREKVKDMARRYSQLSDT
KRRRKQVDLDEVLETINKKKKFGSNYKYPDWDPRFDNCSVEQLTELIQSLERSQTIM
QHRLRAVVESQMITTTTTTTPPMNHLQQHSNQVSMELYNHGIGTLPQLPVSTSAFNQ
AQSLAPIPNSLTIYQNPNMETYSRLRGEQETGTNEFPSTSMVPYNSFNTNCGYPRQHN
QNFGKVEDYSGFRGLQETGINNTNVEDYPRSLWAQGTGMNGLQNMDMYGYNSHSY
DDNGFSHQLVQYPTQQRAAPVFQYMDYPTQDIRPLYEI*
>IiMADS12
MASSSSCFTSSWSPMTKKTNKFPQIRPTLFQKPSSLKATSLTKRRETVFKKAYELSTLC
GIEVCVIYYGSDGELKTWPKDREKVEDMARRYSQLSDMMRRKKRVDLDQFLEKINN
DDSKENKKKKKVKLGSNYKYPDWDPRFDNCSVEQLTVLIQSVERSQTIMQHRLRAV
VESQRQRNMHYMNMANQDQMITTTTPPMNHLQQHSNQLSMEVYNHGIGALQQLPV
STSAFNQAQSLAPIPDSLTIYQNPNMEMYSRSRGVQETGTNEFLSTDILPYNSFNTNYV
NGSPRQFNQNCGNYSGFPVVQETGINNMNVEDYPGSLWEQGTGMNGLQNMDMYG
YNSNSNANDHSLCYVTNYNQLNSLYPYIRTVSYFSRRALEAKKLGVGVCLSVGDDKP
YSNSRILFAMNGFTVRLCGGALSNLIDLNLIHFVSSYVTTPEVSIVSADFFLKFGGSYY
RDKEPEPVRFNAESNASRSSAAAEDQHSGEARTRSRVSQNNDNSYYSEQDETRQQFF
HRGGSSRSYSRSRSRSRSPVYRVRRDAGSSYDRHKTRTRGSPLPSREFNNKRGGDQSS
VYGREDETRKPRETRYHADDFREETMVKGARSSDYDTEFTEDRTRREHLHDGVSDP
RLRRHRSDLTGEKETLRRDGDGGGGFRRSSNIPCKFFAAGICRNDKHCRFSHHVADR
KQPRDNNFYRQDNNNHSGGHNRWNDVERLDNGKFGGMEASRASKGTSETKRNGSS
WIDDMEMSPDWNYAAHNLKKRMKEEPGAGIIIGQSSQIRAVVKDSLVNNYDMYSYG
DNKPMVEKPVADSHQNYTNAVSIAPVQAFNQNHTLMPYQNSLTAGGSQQVIAAADF
SVGLNNPESGKVYQDNHHSMVEKAVPVQSTVTREQIDHISNISATLAQFLANGQPIPQ
LAQALQMPLHSESSMAVQPNQATQSNSASNNLNPLWGLGMSTGADGGPPVAASLVS
NLDGVRELALNAKASEENGDKKTDEASKEEEGKKTGEDDGDAENVVDDDEDDDGS
DEEGKKEKDPKGMRAFKFALVEIVKELLKPAWKEGKMNKDGYKNIVKKVVEKVTG
SMQSGNVPQTQEKIDHYLSASKPKLTKLVQAYISKVKKT*
>IiMADS13
MRSPRSSSRYNSSSSRSRAATRLKKKLLTIFKKAQELTTLCDIEACVIHYGPDGELKTW
PEERDKVRSLALRYIQLSEAKRRKKSVDLSGFLHEKKKTMTTYFEKKAKRKVQELKY
PISDHYSPEQISQMIQSLELSYSVLQERRRFLEAMTNFEDHQQHLQTQSLNPSQFAKCP
RDYSALPQESGLKNQEPCVYDQNNNFQHLCVSHTQGGYDHNMCLSNINNFQHPCVS

NTQDLSALPSELQASLNNYGLNQSMQMALQESVSNYGLMQHELYGFDQNMCMTDI
TNSNVLDPCLSNRVSGDFCFDFQDPYGGNMVGNPSFSQDFPDMSLAPPKRS*
>IiMADS14
MTTTAKNCRAKLSVRKDTIFKKHSSSSLLSSSSSYKRRVTTVLKKAYELSELCGVDVC
IICYDREGNLVKTWPEDEAKVTVMAERFSGLNEQERNKKSTNLSRFLNKKMMEERK
ASLKANDNRFSQKLLEIEDSLVSRLPVFQENPLLLLEHRQDHDHTSADLSSSDDHHQT
SILNHPSTTADLPTTSSSSLNHPSKFSILLYNHDNGTFTQLPNSALPRFGQLPCNQDYGT
NYLDSLLGEQGCTNNSDLPPPLMQTQPPIFQKTDMVD*
>IiMADS15
MTTTAKTCRAKLSVRKDTIFKKHSSSSLLSSSSSSYKRRVTTVLKKAYELSELCGVDV
CIICYDREGNLVKTWPEDEANVRVMTERFSRLSEQERNKKSTNLSRFLNKKMMEERK
ASLKANDNRFSQKLLEIEDSLVSRLPVFQENPLLLLEPGQDHDHTSADLSSSDDHHQT
SILNHPSTTADLPTTSSSSLNHPSKFSILLYNHDNGTFTQLPNSALPSFEQLPYNYLDSL
LGEQGCTNNSDLAPPLMQTQPPIFHKTDMVD*
>IiMADS16
MCASSNSLAATSLRNRLKTIFKKAYELSILCQVEVCVIYYGPNGELKTWPKEREIVRD
RALRYNAAAKRKNSLNLNEFLDSKKKKRTLSTLQEKLCFVEAQKQRNINLVHQSVTP
SSMNQHHHQNQTVQPLNPSQLSLYLYNRDDATLSQLPLSASHSNQLINYQNQSTMQQ
QQRLYDFDHKITNRNFQQPCVSNTQDHTALLSAQESGVKNHLMQQQELYGSRQNMC
MMNTITNSNVLRHPCPTNTVPEEFSFGLQKNPDGDMVGRTSGSQDFPNMCSAYDASR
LLQTSSLPSLHQPIPNSYLLPDNPTCL*
>IiMADS17
MARPKLKFTWIANQSARKSACKKRMQGLMKKADELTILCNMSACLVFFNRDDNKLV
VWPSQEVAKSLIDRFYSLPAIERNMKAEDQESFIRANTKKVEKKLADSRKVENLISYS
SKKISGLARELGYTEQPCTSVNELFLGNEAPRASDVASEWGHGFSSVGSGSIYLMDK
WFPADTNVQEHGDGTHLRPMVSRFDLNLEPSDDDEDMETYEGESSKSGGEDDA*
>IiMADS18
MRTDKLSVRNQIRFKKSSSSSAAKKNTDLSSRERTIFKKAYELETLCDIEVCVLYYGH
DGNLIKTWPEDQSKVRDMAERFHRLSDKEKLKKSTNLSQFLNKKINDERKTSLGTND
SKLSHKFSELEDSLRTQLRIFQNKIPLLQQTEPVQNQSLVASSSSFLSTDGSFSQLADQA
TTSLSTGLLGSQVFHLGSCSNKIDLPAMIPPHQMQAHDPLLNFDQYVASNQTPAFLDP
TMMFSTYN*
>IiMADS19
MRTDKLSVRNQIRFKKSSSSSAAKKNTDLSSRERTIFKKAYELETLCDIEVCVLYYGH
DGNLIKTWPEDQSKVRDMAERFHRLSDKEKLKKSTNLSQFLNKKINDERKTSLGTND
SKLSHKFSELEDSLRTQLRIFQNKIPLLQQTEPVQNQSLVASSSSFLSTDGMSSPVLIND
PLMNLSTSLIHDQSRAASYGFTTTHLSPTLIGHPHQQWTEPLTNHLSGVYSDSFATDSL
NHRQGKFSFFLYNHDEGSFSQLADQATTSLSTGLLGSQVFHLGSCSNKIDLPAMIPPYQ
MQAQDPLLNFDQFIN*
>IiMADS20
MRTDKLSVRNQIRFKKSSSSSAAKKNTDLSSRERTIFKKAYELETLCDIEVCVLYYGH
DGNLIKTWPEDQSKVRDMAERFHRLSDKEKLKKSTNLSQFLNKKINDERKTSLGTND
SKLSHKFSELEDSLRTQLRIFQNKIPLLQQTEPVQNQSLVASPSSFLSTDGMSSPVLIND
PLMNLSTSLIDDQSRAASYGFTTTHLSPTLIGHPHQQWTEPLTNHLSGVYSDSFATDSL

NHRQGKFSFFLYNHDEGSFSQLADQATTSLSTGLLGSQVFHLGSCSNKTDLPAMIPPY
QMQAQDPLLNFDQFIN*
>IiMADS21
MRTDKLSVRNQTRFKKSSSSSAAKKNTDLSSRERTIFKKAYELETLCDIEVCVLYYGH
DGNLIKTWPEDQSKVRDLAERFHRLSDKEKLKKSTNLSQFLNKKINDEKNRSLGTND
SKISHQFLELEDSLRTQLRIFQNKIPLLQQTEPVQNQSLAVSPSSFFSTDGMSSPVLIND
QSMNLTTSLIDDQSRAASYGFTTTHLSPTLIGHPHQQWTEPLTNHLSGVYSDSFATDSL
NHRQGKFSFFLYNHDEGSFSQLADQATTSLSTGLLGSQVFHRGSCSNKIDLPAMIPPH
QMQAHDPLLNFDH*
>IiMADS22
MRTDKLAVRNQIRFKNSAAKKNTDLSSRERTIFKKTYELETLCDIEVCVLYYGHDGNL
IKTWPVDQSKVRDLAERFHRLSDKEKLKKSTNLSQFLNKKINDERKTSLGTNDSKLS
HKFSELEDSLRTQLRIFQNKIPLLQQTEPVQNQSLVASSSSFLSTDGSFSQLADQATTSL
STGTWVASVSSRKLQQQN*
>IiMADS23
MASSSSSSSPTRKKEPKLSVRNETRFKKPSYSWTKNKNLLLREKTILKKAFELSTLCD
NDVCVIHYDRNGELVSTWPEDRSKVRDMAERFSKLSVKEKLKKSTNLSQFLNKKLL
DEKKNIKFTQKVVDFEDQSRLAGISEQNHNFLVSPNFFSKDVPSSSSSSLIDDPLKNLC
PYGYVPDLPQQTGSSTNLLMSGGDVSITSTEQDMSRVVYNSAAATGSSNHQRQGKFS
IFVFNHETATFTQLPDSISSSFDQTYNLKP*
>IiMADS24
MTTTAKNCRAKLSVRKDTIFNKHSSSSLLSSSSSYKRRVTTVLKKAYELSELCGVDVC
IICYDREGNLVKTWPEDEAKVTVMAERFSGLNEQERNKKSTNLSRFLNKKMMEERK
ASLKANDYRFSQKLLEIEDSLFMGDSCSTATAGDAHIKSLKDLYTRKAFLAYISAKKY
LQEGRSTLIQFFGLHFE*
>IiMADS25
MTTTAKTCRAKLSVRKDTIFKKHSSSSLLSSSSYKRRVTTVLKKAYELSELCGVDVCII
CYDREGNLVKTCPEDEAKMMEERKASLKANDNRFSQKLLEIEDSLVSRLPVFQENPL
LLLEHRQDHDHTSADLSSSDHHHQPSILNHPSTTADLPTTSSSSLNHPSKFSILLYNHD
NGTFTQLPNSALPSFGQLPYNQEYGTNYLDSLLGEQGMRSCNNNSDLAPPLMQTQTP
IFQQFDHQFMQTQPVIPSSGYTPTMMFS*
>IiMADS26
MAKGTKRKIEIKKLETKQQRAVTCSKRRPTLFSKAADLCLSSGANIAVFVTSPANKSK
VTDSFSGHSSASEIAQCFLDGTLPPNINPPPKLGFWWTDPDLYHSCNDMTELSIIEDRM
KRAKTDLIACLEKQENPRMSFDHRKPSSSLDEYFGGSSSKIVSGICSGESSSDEQSRYL
VNSDLGCFNCSRETEKENKQIKSLPQEKEPIINPGGGYDDQSFWGELYEENTLDNTSE
EDDCMIDIGEYLSD*
>IiMADS27
MGRKMVKMARITNEKTRITTYRKRKACLYKKANEFSTLCGVNTCLIVYGPSRAGEER
INDPELWPVDESKVREIISKYRDTASSSCTKTYTVQECLEKSKTKVAKVKYCPWDKKL
DNCSLHELYATFVAVGNKIQEAENRNQTYPDASWSTDQLDLFGYNQQQCLEQHQLFP
LSSMEQNGFAFLPFLNQMTSNTGEGASFPNVTEPEMTQAMFYGSCSDGQYAPMVQR
TAYMETMQWGLGNSMFNNVKPFATYPLRFGQVTDLENSVGGLVIRISSSKNVVFL*
>IiMADS28

MGRKMVKMARITNEKTRITTYRKRKACLYKKANEFSTLCGVNTCLIVYGPSRAGEER
INDPELWPTDESKIREIISKYRDTASSSCTKTYTVQECLEKSKTKVAKVKYCPWDKKL
DNCSLHELYATFVAVGNKIQEAENRNQTFPDASWSTDQLDLFGYNQQQCLQQHQLFP
LSSMEQNGFALVPFLNQMTSNTGEVASFPNVTEPEMTEAMFYGSCSDGQYAPMVQRT
AYMETMQWGLGNSMFNNVKPFADYPLRFGQVTDLENSVGGLVITISSSKNVVFL*
>IiMADS29
MGRKMVKMARITNEKTRITTYRKRKACLYKKANEFSTLCGVNTCLIVYGPSRAGEQ
RVNDPELWPVDESKVREIISKYRDTASSSCTKTYTVQECLEKSKTKVAKVKYCPWDK
KLDNCSLHELYATFVAVASLEQHQLFPLSSMEQNGFAFLPFLNQMTSNTGEGASFPNV
TEPEMTQAMFYGSCSDGQYAPMVQRTAYMETMQWGLGNSMFNNVKPFATYPLRFG
QVTELENSGKSPM*
>IiMADS30_IiSHP2
MEGGASNEVAESSKKIGRGKIEIKRIENTTNRQVTFCKRRNGLLKKAYELSVLCDAEV
ALVIFSTRGRLYEYANNSVRGTIERYKKACSDAVNPPSVTEANTQYYQQESSKLRRQI
RDIQNLNRHILGESLGSLNLKELKNLEGRLEKGISRVRSKKHEMLIAEIEYMQKREIEL
QNDNMYLRSKISERAGLQQQESSVIHQGTVYESGVSSSHQSEQYNRNYIPVNLLEPN
QNSSDQNQPPLQLV*
>IiMADS31
MDEGGSSHDVECSKKIGRGKIEIKRIENTTNRQVTFCKRRNGLLKKAYELSVLCDAEV
ALVIFSTRGRLYEYANNSVKGTIERYKKACSDAVNPPSVTEANTQYYQQEASKLRRQI
RDIQNSNRHIVGESLGSLNFKELKNLEGRLEKGISRVRSKKNELLVAEIEYMQKREME
LQHDNMYLRAKVAQGARLNPEQQGSSVLQGTAVYESSLSHDQSQHYNRNYIPVNLL
EPNQQFSGQDQPPLQLV*
>IiMADS32
MGRGRVEMKMIENKINRQVTFSKRRNGLLKKAYELSVLCDAEVALIVFSSRGKLYEF
GSVGVERTIERYHRCYNRSLGNNRPDEASTQNWCQEVTKLKSKYESLVRTNRHLLGE
DLGEMSVKELQALERQLEAALTATRQRKTQVMMEEMEDLRKKERQLGDINKQLKIK
FETEGYAFKTFQDLWSNSAASVAGDPNNSEFPVQSSHLHSVDCNTEPFLQIGFQQDYY
VQGEGSSVSKSNVACSTNFVQNWVL*
>IiMADS33_IiSEP3-2
MGRGRVELKRIENKINRQVTFAKRRNGLLKKAYELSVLCDAEVALIIFSNRGKLYEFC
SSSSMVRTLERYQKCNYGAPEPNVPSREALAELSSQQEYLKLKERYDALQRTQRNLL
GEDLGPLSTKELESLERQLDSSLKQIRALRTQFMLDQLNDLQSKLSDGYQMPLQLNP
NPEDHVDYGRHQQQHSHQAFFQPLECEPILQIGYHQGQQDHGMGAGPSVNNYMLG
WLPYDTNSI*
>IiMADS34
MGRGRIEIKKIENVNSRQVTFSKRRNGLMKKAKELSILCDADVALIIFSSTGKIYDFSS
GCMEQILSRYGYSAAPADHHHKQREQQLLLCSSHENGDVLRKDDSLKSELERLQLAI
ERLKGKELDGMSFSDLISLENQLNDSLHSVKDRKTQVLLNQVERSRLQEKRALEENQ
ILRKQVEMLGRGSSAPKGLSEIPQLSSPQAEPESSSSDDDENDNDDHHSDTSLQLGLSS
SGYCAKRKKPKIESPCDNSGSQVASD*
>IiMADS35_IiSEP4
MGRGKVELKRIENKINRQVTFAKRRNGLLKKAYELSVLCDAEIALLIFSNRGKLYEFC
SSPSGMTKTVEKYRKHSYATMDPNQSAKDLQEKYQDYLKLKSRVEILQHSQRHLLGE

EIAGMGVNELEQLERQVDASLRQIRSTKARSMIDQLSELKTKEEMLLETNRDLKRKL
EESDAALTQSVWGSSSAAAEHSHQQQQQQQQGMSSYQANPPHREAGFFKPLQGNVA
LQMSHYNPGVTNANNSATTSQNVINGFFPGWMV*
>IiMADS36_IiAP1
MGRGRVQLKRIENKINRQVTFSKRRAGLLKKAHEISVLCDAEVALVVFSHKGKLFEY
STDSCMEKILERYERYSYAERQLIAPESDVNTNWSMEYNRLKAKIELLERNQRHYLG
EDLQAMSPKELQNLEQQLDTALKHIRSRKNQLMYDSINELQRKEKAIQEQNSMLSKQ
IKEREKVLRAQQEQWDQQNHGQNMPPPPPQQHQMQHPYMLSHQPSPFLNMGGLY
QEEDPMAMRRNDLDLSLEPVYNCNLGCFAA*
>IiMADS37
MGRGRVQLKRIENKINRQVTFSKRRAGLLKKAHEISVLCDAEVALVVFSHKGKLFEY
STDSCMEKILERYERYSYAERQLIAPESDVNTNWSMEYNRLKAKIELLERNQRHYLG
EDLQAMSPKELQNLEQQLDTALKHIRSRKNQLMYDSINELQRKEKAIQEQNSMLSKQ
VTFVVIIKKCFPLHYY*
>IiMADS38_IiFUL
MGRGRVQLKRIENKINRQVTFSKRRSGLLKKAHEISVLCDAEVALIVFSSKGKLFEYS
TDSCMERILERYDRYLYSDKQLVGRDISQSENWVLEHAKLKARVEVLEKNKRNFMG
EDLDSLSLKDLQSLEHQLDAAIKSIRSRKNQAMFESISALQKKDKALQDHNNTLLKKI
KEREKKTGLQEGQLVQCSNNSSILQPQYCLTSSRDGFVRRVGGEDGGASSLTEPNSLL
PAWMLRPTTNE*
>IiMADS39_IiSEP3
MGRGRVELKRIENKINRQVTFAKRRNGLLKKAYELSVLCDAEVALIIFSNRGKLYEFC
SSSSMVRTLERYQKCNYGAPEPNVPSREALAELSSQQEYLKLKERYDALQRTQRNLL
GEDLGPLSTKELESLERQLDSSLKQIRALRTQFMLDQLNDLQSKERMLTETNKTLRLR
LSDGYQMPLQLNPNPEDHVDYGRHQQQHSHQAFFQPLECEPILQIGYHQGQQDHGM
GAGPSVNNYMLGWLPYDTNSI*
>IiMADS40_IiSEP1
MGRGRVELKRIENKINRQVTFAKRRNGLLKKAYELSVLCDAEVALIIFSNRGKLYEFC
SSSNMIKTLERYQKCSYGSIEVNNKPAKELENSYREYLKLKGRYENLQRQQRNLLGE
DLGPLNSKELEQLERQLDGSLKQVRSIKTQYMLDQLSDLQSKEQMLLETNRALAMK
LDDMIGVRSHHMGGGGEWEGNEQNVSYAHHQAQSQGLYQPLECNPTLQIGYDNPV
CSEQITATTQAQAQPGNGYIPGWML*
>IiMADS41
MGRGRVELKRIENKINRQVTFAKRRNGLLKKAYELSVLCDAEVALIIFSNRGKLYEFC
SSSSMVRTLERYQKCNYGAPEPNVPSREALAVELSSQQEYLKLKERYDALQRTQRNL
LGEDLGPLSTKELESLERQLDSSLKQIRALRVLIFQSRVYIYIYICYFTHTMIENHIS*
>IiMADS42_IiSEP2
MGRGRVELKRIENKINRQVTFAKRRNGLLKKAYELSVLCDAEVSLIVFSNRGKLYEFC
STSNMLKTLERYQKCSYGSIEVNNKPAKELENSYREYLKLKGRYENLQRQQRNLLGE
DLGPLNSKELEQLERQLDGSLKQVRCIKTQYMLDQLTDLQGKEHILLEANRALSMKL
EDMIGVRSHHIGGAWEGGDQQNVGYGHHQAQSHGLFQSLECDPTLQIGYNHPVCSE
QMAVTTQGQSQPGNGYIPGWML*
>IiMADS43
MVRGKTQMKRIENATSRQVTFSKRRNGLLKKAFELSVLCDAEVSLIIFSPKGKLYEFA

SSNMQDTIDRYLRHTKDRISSKPVSEENMQHLKHEAANMMKKIEQLEASKRKLLGE
GIGSCSIEELQQIEQQLEKSVKCIRARKTQVFKEQIEQLKQKEKALAAENEKLAEKWG
SHEIEGWSNKNQESGRGDEESSPSSEVETQLFIGLPCSSRK*
>IiMADS44
MFSAINQKKMIIYIIFSVKGKLNKEKEMVRGKTQMKRIENATSRQVTFSKRRNGLLKK
AFELSVLCDAEVSLIIFSPKGKLYEFASSNMQDTIDRYLRHTKDRISSKPVSEENMQHL
KHEAANMMKKIEQLEASKRKLLGEGIGSCSIEELQQIEQQLEKSVKCIRARKTQVFKE
QIEQLKQKEKALAAENEKLAEKWGSHEIEGWSNKNQESGRGDEESSPSSEVETQLFIG
LPCSSRK*
>IiMADS45
MFSAINQKKMIIYIIFSVKGKLNKEKEMVRGKTQMKRIENATSRQVTFSKRRNGLLKK
AFELSVLCDAEVSLIIFSPKGKLYEFASSNMQDTIDRYLRHTKDRISSKPVSEENMQHL
KHEAANMMKKIEQLEASKRKLLGEGIGSCSIEELQQIEQQLEKSVKCIRARKTQVFKE
QIEQLKQKVKDSSGGIFQLNNVLVLDFYNWNKKTLQEKALAAENEKLAEKWGSHEI
EGWSNKNQESGRGDEESSPSSEVETQLFIGLPCSSRK*
>IiMADS46
MGRGKIVIRRIDNSTSRQVTFSKRRSGLLKKAKELSILCDAEVGVIIFSSTGKLYDYAS
NSSMKSIIERYNKVKEDQHQLLNHASEIKFWQREVATLQQQLHYLQECHRKLIGEELS
GMNANELQNLEDQLETSLKGIRLKKDQLLTDQIRELNRKGQIIQKENQELHSMVDM
MRKENIKLQKKVHGSTDAIEGNSSVDRISNGKETYAPPQLQLIQLQPPREKSIRLGLQL
P*
>IiMADS47
MVRGKTEMKRIENATSRQVTFSKRRNGLLKKAFELSVLCDAEVALIIFSPRSKLYEFST
SSIAKTIERYQKRVKEIGINNKKEDNSQQARGETYGLTKKIEQLEISKRKLLGEGIDACS
IEELQQLENQLERGLTRIRAKKYQLLREEIEKLKEQERNLNKENKELKEKWREMGAIV
ASSTSTLSSSEVNTDDNMEVETGLFIGPPETRQLKKTPQN*
>IiMADS48
MARGKIQLKRIENPVHRQVTFCKRRTGLLKKAKELSVLCDAEIGVVIFSPQGKLFELA
TKGTMEGMIEKYMNCTGGGRGSSSATFTAQEQLQPPNHNPKDEVNVLKQEIEMLQK
GIRYMFGGGDGAMNLEDLLLLEKHLEYWISQIRSAKMEIMLQEIQSLRNKEGVLKNA
NKYLLEKIEENNNNILDANFATVETNYSYPLTMPSEIFQF*
>IiMADS49
MDHNLTFHFLQLLQISYFPENHFPKKNKTFLFVHLPPTANTEYQMELGGDSSPQRKSG
RGKIEIKRIENTTNRQVTFCKRRNGLLKKAYELSVLCDAEVALIVFSSRGRLYEYSNNS
VKGTIERYKKAISDNSNTGSVAEINAQYYQQESAKLRQQIISIQNSNRQLMGETIGSMS
PKELRNLEGRLDRSINRIRSKKNELLFAEIDYMQKREVDLHNDNQLLRAKIAENERNN
PSMNLMPGGSNYEQIMPPPQTQTFDSRNYFQVAALQPNNHHYSSTGRQDQTALQLV*
>IiMADS50
MGRGKIAIKRIDNSTSRQVTFSKRRNGLLKKAKELAILCDAEVGVIIFSSTGRLYDFSSS
SMKSVIERYSEAKGETNSEINPASEIKFWQKEAAILKRQLHNLQENHRKMMGEELSGL
SVEDLQKLENQLELSLRDVRMKKDQMLVEEIQELNREGNLVHQENLDLHKKVNLMR
QQNMELHKKVSEVEGVKSADKNSLLTNGLDMRDNSSEHVHLQLSQPQQHDETRSK
AIQLSYFSFIA*
>IiMADS51

MVRGKIEMKKIENATSRQVTFSKRRNGLLKKAYELSVLCDAQISLIIFSQRGRLYEFSN
SDMQKTIERYRKYTKDHETSNHNSEIYIQQLKEEASHMTRKIELLEVHKRELLGQGLA
SCSVEELQEIDSQLQRSLGKVRARKAQLFREQLEKLKAKEKQLLEENVQLHQKSVM
DPWRRSIDQQEKFKVVDLNLEVQTDLFIGLPERHCK*
>IiMADS52
MAREKIQIRKIDNATARQVTFSKRRRGLFKKAEELSVLCDADVALIIFSSTGKLFEYCS
SSMREVLERHNLQSKNLDKLDQPSLELQLVENSDHARMSKEIADKSHRLRQMRGEEL
QGLNIEELQQLEKALEAGLTRVIETKSEKIMNEISYLQRKGTQLMDENKRLRQQVHIL
PFVFNTYL*
>IiMADS53
MAREKIQIRKIDNATARQVTFSKRRRGLFKKAEELSVLCDADVALIIFSSTGKLFEYCS
SSMREVLERHNLQSKNLDKLDQPSLELQLVENSDHARMSKEIADKSHRLRQMRGEEL
QGLNIEELQQLEKALEAGLTRVIETKSEKIMNEISYLQRKGTQLMDENKRLRQQGTQL
TEENERLGKQIYNNVREGHSSESITNAGNSTGAPVDSESSDTSLRLGLPYGG*
>IiMADS54
MNPEEEEVGNKRKRREMGRGKIEIKKIENQTARQVTFSKRRSGLIKKTHELSVLCDAH
IGLIVFSATGKLSEYCSEPFKMPQLIDRYLQTNGLRLPDPNDDREELCQEMEVLRRETC
KLELRLRPYHGHDLASIPPHELDGLEQQLEHSVLKVRERKNELIQQQLENLSRKRRM
LEEDNNNMYRWLHERRNAIEFQQAGIDTKPGEYQQFLEQLQYYNDHHQQQPNSVLQ
LATLPSEIDPNYHLQLAQPNLQNDPRAKFD*
>IiMADS55
MGRGKIEIKRIENANSRQVTFSKRRAGLLKKAHELSVLCDAEVAVIVFSKSGKLFEFAS
TGMKKTLLRYGNYQSSSDAPLTNSKAEDQKDCREVDLLKDEISKLQEKQLQLQGKG
LNILSLKELQHLEQQLNVSLISVRERKELLLTKQLEESRLKEQRAELENETLRRQVNGT
NSYIFC*
>IiMADS56
MGRGKIEIKRIENANNRVVTFSKRRNGLVKKAKEITVLCDAKVALIIFASNGKMTDYC
CPSMDLGAMLDQYQKLSGKKLWDAKHENLSNEIDRIKKENDSLQLELRHLKGEDIQ
SLNLKNLMAVEHAIEHGLDRVRDHKMEYLVTKRRNEKMIVEENRQLSFQLQQQEMA
IASNARGMMMRDQDGQFGYRVQPIQPNLQEKIMSLVID*
>IiMADS57
MVRGKIEIKKIENTTSRQVTFSKRRSGLFKKAHELSVLCDAQVAAMIFSQKGRLYDFA
SSDIQKTIKRYSEFKREYFVAESHPTEQYVQALKREMVTMVEKIEILEVHNRKLMGQS
LASCSVKELQDIATQIERSLHIIRSRKTKLYGDEVEKLKAKERELQDERVRLCGGVGE
GPMGMPSGSKEKEDVETDLVIGLPKSRP*
>IiMADS58
MGRGKIEIKRIENANSRQVTFSKRRAGLLKKAHELSVLCDAEVAVIVFSKSGKLFEFAS
TGMKKTLLRYGNYQSSSDAPLTNSKAEDQKDCREVDLLKDEISKLQEKQLQLQGKG
LNILSLKELQHLEQQLNVSLISVRERKELLLTKQLEESRLKEQRAELENETLRRQVQEL
RSFLPSINQHSVPSYIKCFAIDPKNPLVNNSSLDDINCSLQKTNSDTTLQLGLPGEAHDR
RKNEGNGESPSSDSVTTSTTGAAAQRIGLV*
>IiMADS59
MGRKKVEIKLIENKSSRQVTFSKRRNGLIEKARQLSILCESSVAVLVVSASGKLYNSAA
GDNMTEIIDRYEVQHADELRNLDLAEKTRNYLPHQELLEFVKSHLEEANAADVSVDS

LSSLEDQLETALSVTRARKTELMMEFVKTLQAKEKLLREENLVLASQMAKTTFQGTE
GDKEMSAASSSGINPRETLSLLK*
>IiMADS60
MAREKIRIKKIDNLTARQVTFSKRRRGIFKKADELSILCDADVALIIFSATGKLFEFSSSR
MRDILGRYNLHASNINKLMGQPSPYHQLENCNLSRLSKEVEDKTKQLRKLRGEDLEG
LNLEELQRLEKSLESGLSRVSEKKGECVMSQISSLEKRGSELVDENRRLREQLVSLEM
AKTMETESATANVSSYDSGAPLEDDFSDTSLKLGLPSWQ*
>IiMADS61
MVRGKIEIKKIENTTSRQVTFSKRRSGLFKKAHELSVLCDAQVAAMIFSQKGRLYDFA
SSDIQKTIKRYSEFKREYFVAESHPTEQYVQALKREMVTMVEKIEILEVHNRKLMGQS
LASCSVKELQDIATQIERSLHIIRSRKTKLYGDEVEKLKAKERELQDERVRLCGGVCTT
WSYHYYLYVHRHRVINTYKLIYTL*
>IiMADS62
MVRGKIEIKKIENVTSRQVTFSKRRSGLFKKAHELSVLCDAQVAAIVFSQSGRLYEFSS
SEMGKTIERYGKFSNEYFVPGRPQVELYLQELEKEMDLMVKKIDQLEVHQRKLMGQ
GLGSCSVAELQEIVTQIEKSLRIVRSRKAELYADQLGKLKEKERVLLDERRRLREKEIR
ERLLRPVLPVTLNTEKGEPEGGCSTKHSSEVETDLFIGLCVSRL*
>IiMADS63
MVRGKIEIKKIENVTSRQVTFSKRRKGLLKKAHELSVLCDAQVAAIVFSQKGRLYDFA
SCDMQKMMERCEIHRREYFGAETLQKQQYVQELKNEMAIMVDKIELLQLHCRKLM
GQDLDSCSVEELKEITTRIEKSLTIIRSRKAKLNEDKIEKLKAEMAKEKEVFNEGSRLR
QMFEEQPLWTQSRSLESEKSAPSYGCGNMNISDVGTDLSIGLPESRV*
>IiMADS64
MVRGKIEIKKIENGTSRQVTFSKRRSGLFKKAHELSVLCDAQVAAIVFSQSGRLYEFSS
SEMEKTIERYRKYSNDYFVPGSPQVELYLQELKKEVDIMVKKIDQLEVHQRKLMGQG
LGSCSVAELQEVDTQIEKSLCIVRSRKAELYADQLGKLKEEERELLDERRRLCEKEREL
LNERRRLREQEIHTEKDEPEGGCKTKHSTEVETDLFIGLPVTRL*
>IiMADS65
MVRGKIEIKKIENVTSRQVTFSKRRSGLFKKAHELSVLCDAQVAAIVFSQSGRLYEFSS
SEMGKTIERYGKFSNEYFVPGRPQVELYLQELEKEMDLMVKKIDQLEVHQRKLMGQ
GLGSCSVAELQEIVTQIEKSLRIVRSRKVQLQNAYIYIWV*
>IiMADS66
MARGKIQIKRIENQTNRQVTYSKRRNGLFKKAHELTVLCDARVSIIMFSSSNKLHEFIS
PNTTTKEIIDLYQTVSDVDVWSAHYERMQETKRKLLETNRNLRTQIKQRLGECLEEL
DIQELHSLEEEMENTNKLVRERKFKSLGNQIETTKKKNKSQQDIQKNLIHELELRAED
PHYGLVDNGGDYDSVLGYQIEGSRAYALRFHQNHHPHYPNHALHAASASDIITFHLL
E*
>IiMADS67
MARGKIQIKRIENQTNRQVTYSKRRNGLFKKAHELTVLCDARVSIIMFSSSNKLHEFIS
PNTTTKEIIDLYQTVSDVDVWSAHYERMQETKRKLLETNRNLRTQIKQRLGECLEEL
DIQELHSLEEEMENTNKLVRERKFKSLGNQIETTKKKVTSSIFRSLLHFIIFCLNSFV*
>IiMADS68
MAKKYYGRRKVEMLKMRNETNLQVTFSKRRSGLFKKASELCTLCDARVALVVFSPS
GKVFSFGHPSVEVLFDRLTRRSQNSIPSHSHSTEVERNTNSDLQVLNKILAQVLVEKEK

EKETNKKYKIIKKERLKNNSENWYKNPPEELSLTQAIHMKHVAEDLKEKIDNLRSEFQ
LRIIPHQNYYVTSSSNVVLVPGAVHRGDNYTTHNLFDQNRRLVTSGFSYDPSMTTPSQ
WLLFGYNHDNNNISERIVPDSAVNRPPWYNHIKLDPKSEV*
>IiMADS69
MMMSKKKESIGRQRIPMVKIKKESHRQVTFSKRRAGLFKKASELCTLCGAEIGIIVYS
PAKKPFSFGHPSVEAVFDRYLSRNDLSLAKTQQPQGNAAASCELNMCWTQILSEVEE
EKKKGQAMVQMRKANASRSMINWWEGPVEEMNMVQLHEMKSALEELRK*
>IiMADS70
MMSKKKETMGRQKIPMVKIKKETHRQVTFSKRRAGLFKKASELCTLCGAEIGIIVYSP
AKKPFSFGHPSVESVLDRYLSRNDIVPSLAQTQKPQGNSAGNCELNMRLTQIVSQVEE
EKKKGQAMSEVRKANAERRSMTNWWEGPVEGLNMVQLQEMKFALEELRKIVVSEI
NTAPCKEVKEDVFGFLDDNNVTAPSYMNMSTGKVGEES*
>IiMADS71
MTAKKKGNTGRQKIRMAKIEKESHRQVAFSKRRAGLFKKASELCTLCGVEIAIIVFSP
AKKPFSFGHPSVYSVLDRYRNNSSSPQPRQPHENTAARSELSLVLSQVEEEKKKGDEM
KKASEMVKWSVEEMSLLQLHEMKSALQELRNTIGVPPYSNMNMSTVSIF*
>IiMADS72
MGRRKIKMEKVQDTNTKQVTFSKRRLGLFKKASELATLCNAEVGIVVFSPGNKPYSF
GKPNFDSIAERFKNDSEESDSCEISGHSRVNRARQEKKICKRLNSIIEEVESEKKRGENL
QNWLESAGEDKFNKPIEELSLEDLKEYEAKLKNMHAGIQDEEYGKAKRKTDVQRW
KLEPLTRSTFIQ*
>IiMADS73
MGRRKVTHQLISDNSTRRVTFRKRKDGLLKKLKELTILCGLRACAIIYSDYKEGAEV
WPNRKERRSWRKSRSIAVQWILG*
>IiMADS74
MTRKKLNLAYITNDSMRKSTYNKRKKGFFKKIHELSVLCGIEACAVIYSPFSTSPEVW
PSKSGVKNVVEKFEKVPEMEQEKKMVNHEGFLRQSITKARENNNKKMKENKEKAL
KEAMFQILGGIGDIFKLTDRNREELSANAMPHPAAIAEVGSSSFPKPDVFNSPQLLSEL
WALRPMISSGQRPEQINTFGDNFLPAANDQQGYYQMMNPVGVYDQHPNLNLNHNQ
YQHKIEENPIVAQDEIYNQSQNPNHQQEEWLVNQMMNHPHQMRFPVTDGSNRYYHH
QS*
>IiMADS75
MAKSRVKLSLIADNASRRATFRKRKKGMLKKLNELATLCGVKACAVVYSGQNDSNP
EAWPSREGAEKVLSDFMEVPEMERGKKMYNQQSLTWERIKKEQAKLKRLHEENREL
ELREFMFDLVEGKMPLEYHYYDENVIRDLSSFVDHYVNQLTHRVQTLQANGEPMPFP
PLDVAVADAAPPPPAAGYVNHVAVSDVASVTTTPAGFYDLIQQYQNMNMNESEQAPA
DSSDHVQYQQELAQYQNQHRFYGWNQDMSLNHDLAQSSNQYPNQHQSFMNQWM
APQPQQMSELASVASMDDNYYRYHQLPTASHMPSTSTTTVDLSAPNTKKNVWPTRIG
LN*
>IiMADS76
MRGKKTKGKQKIDIKRVEKKEDRMVTFSKRRNGIYTKLSELSVLCGADVGFLIYSGA
GKPFTFGSPSFDAVAERFLHGIHHPQMINGEGSSSSSLSSSIVDAHKKVRMDELCKNFN
KLMEETDAEEEKRKMSEAAYQPSLPVKSNAWWKVEPNDDEEAKQLLARIKS*
>IiMADS77

MKRTKGKQKIEMKKVEAYGDRMITFSKRKSGIFKKMNEIVALCDMEAAFLVFSQAG
KPYTFAHPSNEEAFGRVKSTLRHEPSAKDDTNTIPLVEAYKRQRIEDLMKKYADLVEE
LEMEKEKEKILKESESEKKLDKMWWNIPAEGLTVEELKRRHQAFLELHVSLCGMAL
QWLGKDGDGSSSDLEGREHCDLVIGLVMRVIQMNHETIHQRPLDSIRDAKYDTASRW
KDSHSGQHVE*
>IiMADS78
MGMMKKVKELSTLCGVNACAIVYSPYDSEPQVWPSTSGVQSVISDFRALPEVEQRK
KMVNHETFVRKMLAKALEHVKRKRKDNRELEMTEVMFQSLMGKMRMFHLNIIDLN
DLGYLIEQYVRDLTHRMNVLENYGTEIGESSNAAAARAPSDGTGTMAYVAPTTAPGA
MIYEVGSSSSSAADVAFFNPIQGQHHHQQFHIPAAPNVGLYEQPMNLYHNQNQQQWF
TETMNRAAEQMGHPIMDGNHHNQQQQQHIQGDSSTTQADYDYGC*
>IiMADS79
MTRKKLNLAYITNDSMRKSTYNKRKKGFFKKINELSVLCGIEACAVIYSPFSTSPEVW
PSKSGVKNVVEKFEKVPEMEQEKKMVNHEGFLRQSITKARENNNKKMKENKEKAL
KEAMFQILGGIGDIFKLTDRNREELSANAMPHPAAIAEVGSSSFPKPDVFNSPQLLSEL
WALRPMISSGQRPEQINTFGDNFLPAANDQQGYYQMMNPVGVYDQHPNLNLNHNQ
YQHKVEENPIVAQDGIYNQSQNPNHQQEEWLVNQMMNHPHQMRFPMMDGSNRYY
HHQS*
>IiMADS80
MGRKKLELAYISNDIVRKATFSSRHEVLIKKLEDLKLICGVDACAIIYNGFDSTPEVWP
SHSGAKEVIEKFVTLTETERSFKSVTHEEFINQEITKVRNKRQQLAEENKEGYLNELM
FGCLSGNMRDLSMNDDDNPELCSFIDQYVKKLTQYKNKTLNTPHFEIGESSSTSMAD
QAGQEHVNSLVSNHVDVPTSNSPQVTSEMQLVISSDQTLAHDTSVASSDQEVYVPVM
NQDEFYNMDQSQNEQEGFVEEMLKLGEQTGFPWMEEDNQF*
>IiMADS81
MTRQKVKMAYIENESSRKSTYKKRKRGILKKAYELATLCDVPIAVMIESPYDSSPEVY
PSREAVEKVVSQWQTLSTMDKSKKMVNQETYLQQRISKATESWRKLGKENRELAM
KEYLKDLNRRVEILTKNDGSSSSSVPAAAAATSVAMPILEMGSSSTGFYDRLREQMQY
NLNMKQTMKDLDLNLKQW*
>IiMADS82
MGRVKLKIKRLESTGNRQVTFSKRKSGILKKAKELSILCDIDIVLLMFSPTGKPTVFHG
EHSCIEEVISKFAQLTPQERTKRKLESLEALKKTFKKLDHDVNIHEFLGARNQTIEGLS
NQVAIYQAQLMECHRRLSCWTNIDRIENTEHLNLLEESLRKSVERLQIHKTVSFTMQE
HYGKNQVLPIECTTTQFHPGIQLPLAMGGNNSMQEAHSMSWLPDNGNQQTILPGDSS
FLPHR*
>IiMADS83
MGRVKLKIKRLESTGNRQVTFSKRKSGILKKAKELSILCDIDIVLLMFSPTGKPTVFHG
EHSCIEEVISKFAQLTPQERTKRKLESLEALKKTFKKLDHDVNIHEFLGARNQTIEGLS
NQVAIYQAQLMECHRRLSCWTNIDRIENTEHLNLLEESLRKSVERLQIHKVCNLIFLST
QSYRHDSGFLFSFVNPAHIDIGYKKDGFLHYAGTLRKEPSFANRMYNDTVSPRDTVAF
GDGR*
>IiMADS84
MGRVKLKIKRLESTGNRQVTFSKRKSGILKKAKELSILCDIDIVLLMFSPTGKPTVFHG
EHSCIEEVISKFAQLTPQERTKRKLESLEALKKTFKKLDHDVNIHEFLGARNQTIEGLS

NQVAIYQAQLMECHRRLSCWTNIDRIENTEHLNLLEESLRKSVERLQIHKEHYGKNQ
VLPIECTTTQFHPGIQLPLAMGGNNSMQEAHSMSWLPDNGNQQTILPGDSSFLPHRE
MDGSIPVYPNCFFESMKQEDQICSNPGQQFEQLEQQGDGCLGLQQVGEEYSYPAPFN
TTLGMEEDQEKMKTEMELNNLQLQQQQQDPSSMYDPTANNSGCFQIPHDQPMFATD
HHHHQHHHHHHQNWVPDAMFGQTSYNQQPN*
>IiMADS85
MGRVKLKIKRLESTGNRQVTFSKRKSGILKKAKELSILCDIDIVLLMFSPTGKPTVFHG
EHSCIEEVISKFAQLTPQERTKRKLESLEALKKTFKKLDHDVNIHEFLGARNQTIEGLS
NQVAIYQAQLMECHRRLSCWTNIDRIENTEHLNLLEESLRKSVERLQIHKTVSFTMQE
HYGKNQVLPIECTTTQFHPGIQLPLAMGGNNSMQEAHSMSWLPDNGNQQTILPGDSS
FLPHREMDGSIPVYPNCFFESMKQEDQICSNPGQQFEQLEQQGDGCLGLQQVGEEYS
YPAPFNTTLGMEEDQEKMKTEMELNNLQLQQQQQDPSSMYDPTANNSGCFQIPHDQ
PMFATDHHHHQHHHHHHQNWVPDAMFGQTSYNQQPN*
>IiMADS86
MGRVKLKIKKLESINARQATYCKRKNGIIKKAKELSILCDIDVLLLMFSPAGKPSLCSG
KHSIGDVIAKFAQLPPQERAKRKLENLEALKKTFLKLEHDVNISEFLKRRCVLIVLSEK
VRFLQTHLSETHARLSYWTEVEKVDSIDDLQQLENSVRQSLYQIRVHKENMLHQQQQ
QLVSTECKTQMQSEIDLDFGMDMEQQLENFSWVRTDENMNVPKKEDDPNLQFYYT
YRDITCSASSSLESYSGLLGKSSDIKTAKLDTSGISGTLADPNQQFSNLSFLNDPKLQQ
LAEWNLLGSPADYYVSQILEASYRPQFGGNWPSSETLTYPVNVFDDPLFSHPNL*
>IiMADS87
MVKRSGGGTKRKIKIEKIAKKESLATGFTKRRNGLYSKVSQLCLLSDAQIAVLATPSSS
HSNVSFFSFGHSSVDAIVTAYLTGQRPVSLPSADREDLGIIMARKELGLGLWWEDELL
ASSKNAAELTEAINSMSTLLQRIREFRSGEADEHETL
>IiMADS88
MVKRGGTKRKIEMKKITCAASASVTLSKRRSGLYSKVSELCLLTDAQVAVLSTPLSSN
SNCHFFSFGHTSVDSIVTAFLNGERHVRVEATKRESDDIGICFARHQLGLPLWCDDDRL
VRSNNLEELTDAINSVSRMLANIEELRSGDALKHQAFANDEDNEPLMNNNETTDQTL
VMSDKTLNFRSDSQIPCILDVPKLLNYVDDYNKNVMTEEQDQIMAICESFSVPDKNN
NSSF
>IiMADS89
MTRKKVKLAFIANDSSRKATFKKRKKGLIKKVNELSTLCGITACAIIYSPYDSNPEVW
PSNSGVQRIISDFRTLPEMDQHKKMVDQETFLRQRIAKASEHLKRQRKDNREMEMTE
VMFQCLVGNMGMFHLNIMDLNDLGYLIDQYLKDVNRRMEILGNSGVELGESSNDAV
AAAPSEATGTLSLVASTTAPATHHHNHHQQQQQLFRHPAAPHGALYEQPRSLNLNHN
QNQQQWFMEMMNQQNHPEQMSYAAEQMGFPFMNDNRH
>IiMADS90
MAKSRVKLSLIADNASRRATFRKRKKGMLKKLNELATLCGVKACAVVYSGQNDSNP
EAWPSREGAEKVLSDFMEVPEMERGKKMYNQQSLTWERIKKEQAKLKRLHEENREL
ELREFMFDLVEGKMPLEYHYYDENVIRDLSSFVDHYVNQLTHRVQTLQANGEPMPFP
PLDVAVADAAPPPPAAGYVNHVAVSDVASVTTTPAGFYDLIQQYQNMNMNESEQAPA
DSSDHVQYQQELAQYQVPPANVSDHIQYQNMAHEEQRQYQPLANVYDQNQHRFYG
WNQDMSLNHDLAQSSNQYPNQHQSFMNQWMAPQPQQMSELASVASMDDNYYRY
HQLPTASHMPSTSTTTVDLSAPNTKKNVWPTRIGL

>IiMADS91
MTAKKKGNTGRQKIRMAKIEKESHRQVAFSKRRAGLFKKASELCTLCGVEIAIIVFSP
AKKPFSFGHPSVYSVLDRYRNNSSSPQPRQPHENTAARSELSLVLSQVEEEKKKGDEM
KKASEMVKWSVEEMSLLQLHEMKSALQELRNTIGVPPYSNMNMSTGLSPIYNSGYG
HAPS
>IiMADS92
MGLKKIEIKYIQDRITRQVTFSKRRIGMFKKADALAKLSNVEVAVLLISPSTGRPYVFG
SPCFNSVVGRFQYPYLKRTEKSSFSPKQIRILKSQSKLDRLMEELKQHKEREKGLKKR
KEEILEKYDIKKIVDLKLEELTAFKEKLEAFRDDMKRKHMEMEASSSLLILSKT
>IiMADS93
MTRKKVKLAFIANDSSRKATFKKRKKGLLKKVNELSTLCGINACAIIYSPYDSNPEVW
PSTSGVERIVSDFRALPEMDQNKKMVDQETFLRQRIAKASEHLKRQRKDNRELEMTE
VMFQCLVGNMGMFHLNIMDLNDLGYLIEQYLKDVNRRMEILGNSEMEIGDSSNTVA
NASPSEAIGSLALV
>IiMADS94
MVRGKTEMKRIENATSRQVTFSKRRNGLLKKAFELSVLCDAEVALVIFSPRGKLYEFS
SSSSITKTVERYQKRIQDLGSNHNRDDNSQQAKGETYGLARKIEQLEISKRKFMGEGL
DASSIEELQQLENQLDRSLTKIRAKKYQLLREEIERLKEKETNLTAENQMLTEKYEME
RGGIIARTSSSTLSEDLDTEDSEMEVVTDLFIGPPETRHSKKFPP
>IiMADS95_IiAG
MGTFHFLQLLQISYFPENHFPKKNKTFLFVHLPPTANTAYQMELGGDSSPQRKSGRGK
IEIKRIENTTNRQVTFCKRRNGLLKKAYELSVLCDAEVALIVFSSRGRLYEYSNNSVKG
TIERYKKAISDNSNTGSVAEINAQYYQQESAKLRQQIISIQNSNRQLMGETIGSMSPKE
LRNLEGRLDRSINRIRSKKNELLFAEIDYMQKREVDLHNDNQLLRAKIAENERNNPS
MNLMPGGSNYEQIMPPPQTQTFDSRNYFQVAALQPNNHHYSSTGRQDQTALQLV
>IiMADS96_IiAGL1
NCVKGTIERYKKACSDAVNPPSVTEANTQYYQQEASKLRRQIRDIQNSNRHIVGESLG
SLNFKELKNLEGRLEKGISRVRSKKNELLVAEIEYMQKREMELQHDNMYLRAKVAQG
ARLNPEQQGSSVLQGTAVYESSLSHDQSQHYNRNYIPVNLLEPNQQFSGQDQPPLQLV
>IiMADS97_IiSTK
MGRGKIEIKRIENSTNRQVTFCKRRNGLLKKAYELSVLCDAEVALIVFSTRGRLYEYA
NNNIRSTIERYKKASSDNTNTHSVQEINAAYYQQESAKLRQQIQTIQNSNRNLMGDSL
SALSVKELKQVENRLEKAISRIRSKKHELLLAEIENLQKREIELDNESIYLRTKIAEVER
FQQHHHQMVSGTEMTAIEVLASRNYFSHSIMTTGSGSGAGHGCSYSDPDKKIHLG
>IiMADS98_IiSVP
MAREKIQIRKIDNATARQVTFSKRRRGLFKKAEELSVLCDADVALIIFSSTGKLFEYCS
SSMREVLERHNLQSKNLEKLDQPSLELQLVENSDHARMSKEIADKSHRLRQMRGEEL
QGLNIEELQQLEKALEAGLTRVIETKSEKIMNEISYLQSKGTQLMDENKRLRQQGTQL
TEENERLGKQIYNNVRERGDGVESENTAVYEEGHSSESITNAGNSTGAPVDSESSDTS
LRLGLPYGG
>IiMADS99
MGRGRIEIKKIENVNSRQVTFSKRRNGLMKKAKELSILCDADVALIIFSSTGKIYDFSS
GCMEQILSRYGYSAAPADHHHKQREQQLLLCSSHENGDVLRKDDSLKSELERLQLAI
ERLKGKELDGMSFSDLISLENQLNDSLHSVKDRKTQVLLNQVERSRLQEKRALEENQ

ILRKQVEMLGRGSSAPKGLSEIPQLSSPQAEPESSSSDDDENDNDDHHSDTSLQLGTA
NKGPTFCYNLLRSLKTYVFVFLRLSSSGYCAKRKKPKIESPCDNSGSQVASD
>IiMADS100
MGRGKIEIKRIENANSRQVTFSKRRAGLLKKAHELSVLCDAEVAVIVFSKSGKLFEFAS
TGMKKTLLRYGNYQSSSDAPLTNSKAEDQKDCREVDLLKDEISKLQEKQLQLQGKG
LNILSLKELQHLEQQLNVSLISVRERKELLLTKQLEESRLK
>IiMADS101
MVGTGKRERIAIRRIDNLAARQVTFSKRRRGLFKKAEELSILCDAEVGLVVFSATGKL
FHFASSSMKQVIDRYDSHSKTLQRSEPQSSQLQSHMDDGTCARLKEELAETSLKLRQ
MRGEELQRLSVEQLQELEKTLESGLGSVLKTKSQK
>IiMADS102
MGRGKIVIQRIDDSTSRQVTFSKRRKGLIKKAKELAILCDAEVGLIIFSSTGKLYDFASS
SMKSVIDRYNKSKIEQQQLLNPASEVKFWQREAAVLRQELHALQENHRQIMGEQLNG
LSVNELNNLENQLEISLRGIRMKKEQTLTHEIQELSQKRNLIHQENLELSRKVQRIHQE
NVELYKKAYTASTNGFIHRELAIPDDESHTQIRLQLSQPEHSDYETPPRGSE
EFSEKAAMGRKKVEIKLIENKSSRQVTFSKRRNGLIEKARQLSILCESSVAVLVVSASG
KLYNSAAGD
